# Investigation of PTC124-mediated translational readthrough in a retinal organoid model of AIPL1-associated Leber congenital amaurosis

**DOI:** 10.1016/j.stemcr.2022.08.005

**Published:** 2022-09-08

**Authors:** Amy Leung, Almudena Sacristan-Reviriego, Pedro R.L. Perdigão, Hali Sai, Michalis Georgiou, Angelos Kalitzeos, Amanda-Jayne F. Carr, Peter J. Coffey, Michel Michaelides, James Bainbridge, Michael E. Cheetham, Jacqueline van der Spuy

**Affiliations:** 1 UCL Institute of Ophthalmology, London EC1V 9EL, UK; 2 Moorfields Eye Hospital NHS Foundation Trust, London EC1V 2PD, UK

**Keywords:** induced pluripotent stem cells, inherited retinal degeneration, retinal organoids, ROs, CRISPR-Cas9, gene editing, translation readthrough-inducing drug, TRID, renal epithelial cell reprogramming, Leber congenital amaurosis, LCA

## Abstract

Leber congenital amaurosis type 4 (LCA4), caused by *AIPL1* mutations, is characterized by severe sight impairment in infancy and rapidly progressing degeneration of photoreceptor cells. We generated retinal organoids using induced pluripotent stem cells (iPSCs) from renal epithelial cells obtained from four children with *AIPL1* nonsense mutations. iPSC-derived photoreceptors exhibited the molecular hallmarks of LCA4, including undetectable AIPL1 and rod cyclic guanosine monophosphate (cGMP) phosphodiesterase (PDE6) compared with control or CRISPR-corrected organoids. Increased levels of cGMP were detected. The translational readthrough-inducing drug (TRID) PTC124 was investigated as a potential therapeutic agent. LCA4 retinal organoids exhibited low levels of rescue of full-length AIPL1. However, this was insufficient to fully restore PDE6 in photoreceptors and reduce cGMP. LCA4 retinal organoids are a valuable platform for *in vitro* investigation of novel therapeutic agents.

## Highlights

• Retinal organoids generated from renal epithelial derived iPSCs from affected individuals to model LCA4
• Correction of *AIPL1* nonsense mutation in LCA4 iPSCs utilizing CRISPR-Cas9
• PTC124-mediated translational readthrough rescues full-length AIPL1 in LCA4 organoids
• Levels of AIPL1 recovery insufficient for full rescue of disease phenotype

## Teaser

Leung et al. isolated renal epithelial cells from affected children to model severe *AIPL1*-associated retinal disease through iPSC retinal organogenesis. Although PTC124-mediated translational readthrough of a common *AIPL1* nonsense mutation rescued low levels of full-length AIPL1 in retinal organoids from affected individuals compared with CRISPR-Cas9-repaired isogenic organoids, this was insufficient to fully rescue the AIPL1 loss-of-function disease phenotype.

## Introduction

Leber congenital amaurosis (LCA), the most severe form of inherited retinal degeneration (IRD), is characterized by early and progressive severe loss of vision within the first few years of life (den Hollander et al., 2008). LCA is genetically heterogeneous, with 26 genes associated with the disease (Retinal Information Network: https://sph.uth.edu/retnet), and is typically inherited in an autosomal recessive manner. Biallelic mutations in the aryl hydrocarbon receptor-interacting protein-like 1 (*AIPL1*) gene (LCA type 4; MIM: 604392) account for 5%–10% of LCA (Dharmaraj et al., 2004; Sohocki et al., 2000).

The 384-amino-acid protein AIPL1, exclusively expressed in retinal photoreceptors and the pineal gland (Sohocki et al., 2000; van der Spuy et al., 2002, 2003), has an N-terminal FK506-binding protein (FKBP)-like domain, followed by a tetratricopeptide repeat (TPR) domain and a C-terminal primate-specific proline-rich domain (PRD). AIPL1 is a specialized molecular co-chaperone that, together with HSP90, enables correct folding and assembly of the cyclic guanosine monophosphate (cGMP)-specific phosphodiesterase 6 (PDE6), a critical enzyme in the phototransduction cascade that hydrolyses cGMP in photoreceptors upon light stimulation (Hidalgo-de-Quintana et al., 2008; Sacristan-Reviriego and van der Spuy, 2018). Studies of the mouse retina revealed that, with a reduction or absence of AIPL1, cone and rod PDE6 levels decrease (Kirschman et al., 2010; Liu et al., 2004; Ramamurthy et al., 2004), and PDE6 subunits are misassembled and targeted to proteasomes for degradation (Kolandaivelu et al., 2009). As a result of reduced PDE6 levels, cGMP accumulates, leading to rapid photoreceptor degeneration in the knockout mouse model (Ramamurthy et al., 2004). Currently, there is no cure or treatment for AIPL1-associated LCA (LCA4).

Nonsense variations giving rise to premature termination codons (PTCs) are found extensively in genetically transmitted disorders, including LCA4 (Mort et al., 2008). *In vitro* expression of *AIPL1* c.94C>T, p.R32X; c.216G>A, p.W72X; c.264G>A, p.W88X; c.487C>T, p.Q163X; c.582C>G, p.Y194X; c.665G>A, p.W222X; and c.834G>A, p.W278X resulted in non-functional truncated protein products, confirming their disease-causing status (Sacristan-Reviriego et al., 2017, 2020). Translational readthrough-inducing drugs (TRIDs) promote ribosomal misreading of PTCs and restore production of full-length proteins (Dabrowski et al., 2018; Ng et al., 2021; Peltz et al., 2013; Roy et al., 2016). PTC124 (ataluren or Translarna) is the only TRID that has been authorized for clinical use in Duchenne muscular dystrophy (DMD) (Campbell et al., 2020). PTC124, with minimal ocular side effects, has also been investigated as a potential TRID in several hereditary ocular disorders, including Usher syndrome (Goldmann et al., 2012; Samanta et al., 2019), choroideremia (Moosajee et al., 2016; Torriano et al., 2018), retinitis pigmentosa (Ramsden et al., 2017; Schwarz et al., 2015; Vössing et al., 2020), LCA (Shahi et al., 2019), and congenital aniridia (Gregory-Evans et al., 2014; Liu et al., 2020; Wang et al., 2017). These findings suggest that TRIDs could rescue nonsense AIPL1 mutations causing LCA.

Reprogramming somatic cells derived from affected individuals into induced pluripotent stem cells (iPSCs) has revolutionized the study of genetic diseases in the last decade (Takahashi et al., 2007). Likewise, investigation of human eye development has benefitted from advances in stem cell differentiation toward three-dimensional (3D) retinal organoids (ROs) (O’Hara-Wright and Gonzalez-Cordero, 2020). This technology provides an opportunity for modeling different IRDs *in vitro* and testing personalized treatments. In this study, we isolated renal epithelial cells from four children with LCA4 harboring a common nonsense variation in the *AIPL1* gene. We developed the first model of *AIPL1* LCA ROs derived from renal epithelial cells from all four affected individuals and investigated the effect of PTC124 in ROs derived from two affected individuals.

## Results

### Generation and characterization of iPSCs from individuals with LCA4

**Figure 1:**
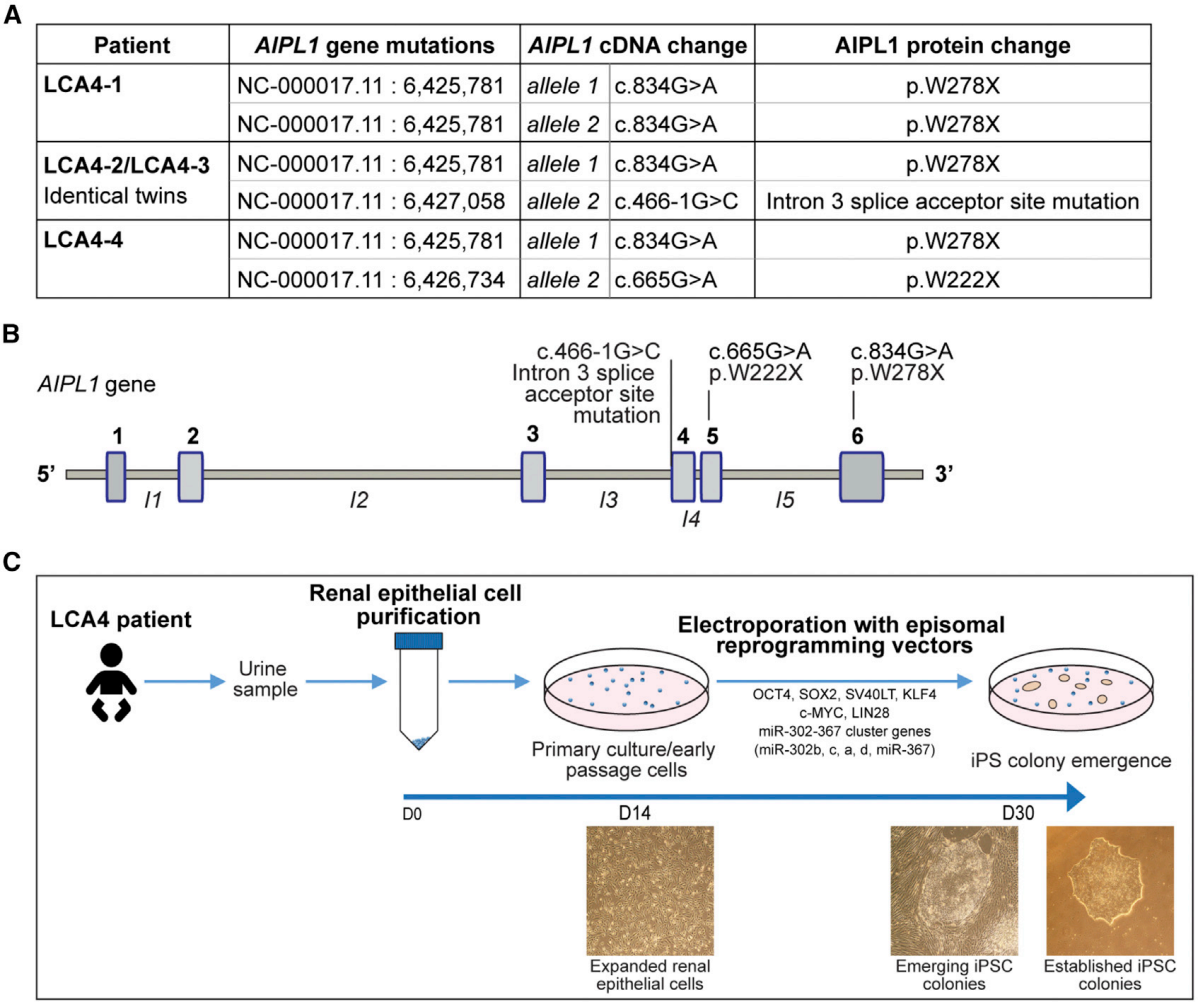
Characterization of LCA4 mutations and iPSC generation from RE cells from affected individuals (A) Summary of the *AIPL1* mutations (genomic site, transcript change, predicted protein change) in individuals with LCA4. (B) Schematic of the *AIPL1* gene; the LCA4 mutation sites are highlighted. (C) Schematic and bright-field images detailing the timeline of iPSC generation from RE cells from affected individuals. Representative bright-field images of RE cells and iPSC cultures from LCA4-1, LCA4-2, LCA4-3, and LCA4-4 are shown in Figure S2A.

Renal epithelial (RE) cells were purified from urine samples from 4 different children with LCA4, aged 2–4 years (LCA4-1, LCA4-2, LCA4-3, and LCA4-4) (Figure 1). LCA4-1 is homozygous for the AIPL1 nonsense mutation c.834G>A, p.W278X (Figures 1A and S1A). LCA4-2 and LCA4-3 are compound heterozygous for c.834G>A, p.W278X and c.466-1G>C, a mutation in intron 3 that abolishes the putative intronic splice acceptor site. LCA4-4 is compound heterozygous for c.834G>A, p.W278X and c.665G>A, p.W222X (Figures 1A and S1A). The locations of these mutations in the *AIPL1* gene are highlighted in Figure 1B.

Clinical investigation showed that LCA4-1 had nystagmus from birth and poor visual function. On examination at 3.2 years of age, the child had a best corrected visual acuity (BCVA) of perception of light in both eyes with bilateral roving eye movements and horizontal and vertical nystagmus. Optical coherence tomography (OCT) revealed residual foveal outer retinal structure (Figure S1B). LCA4-2 and LCA4-3 are monozygotic twins born from non-consanguineous parents and were symptomatic at birth. Electroretinograms (ERGs) performed at the age of 10 months showed extinguished photopic and scotopic responses in both infants and BCVA was light perception for both. OCT performed at 2 years of age revealed residual foveal outer retinal structure (Figures S1C and S1D). The clinical findings for LCA4-4 at 3 years and follow-up at 5 years of age have been described previously (Sacristan-Reviriego et al., 2020).

Multiple iPSC lines from all four LCA4 RE cells derived from affected individuals were generated as described previously (Figures 1C and S2A) (Zhou et al., 2012). iPSC lines from a well-characterized control (CTL) (Parfitt et al., 2016) were expanded in parallel. iPSC cultures from CTL and LCA4 lines uniformly expressed pluripotency markers (OCT4, NANOG, TRA1-80, TRA1-61, and SSEA4) (Figure S2B). Gene expression analyses of iPSCs toward ectodermal, mesodermal, and endodermal lineages confirmed expression of the appropriate lineage markers, demonstrating trilineage differentiation potential (Figure S2C).

### Characterization of RO structure and retinal cell populations from LCA4 iPSCs

**Figure 2:**
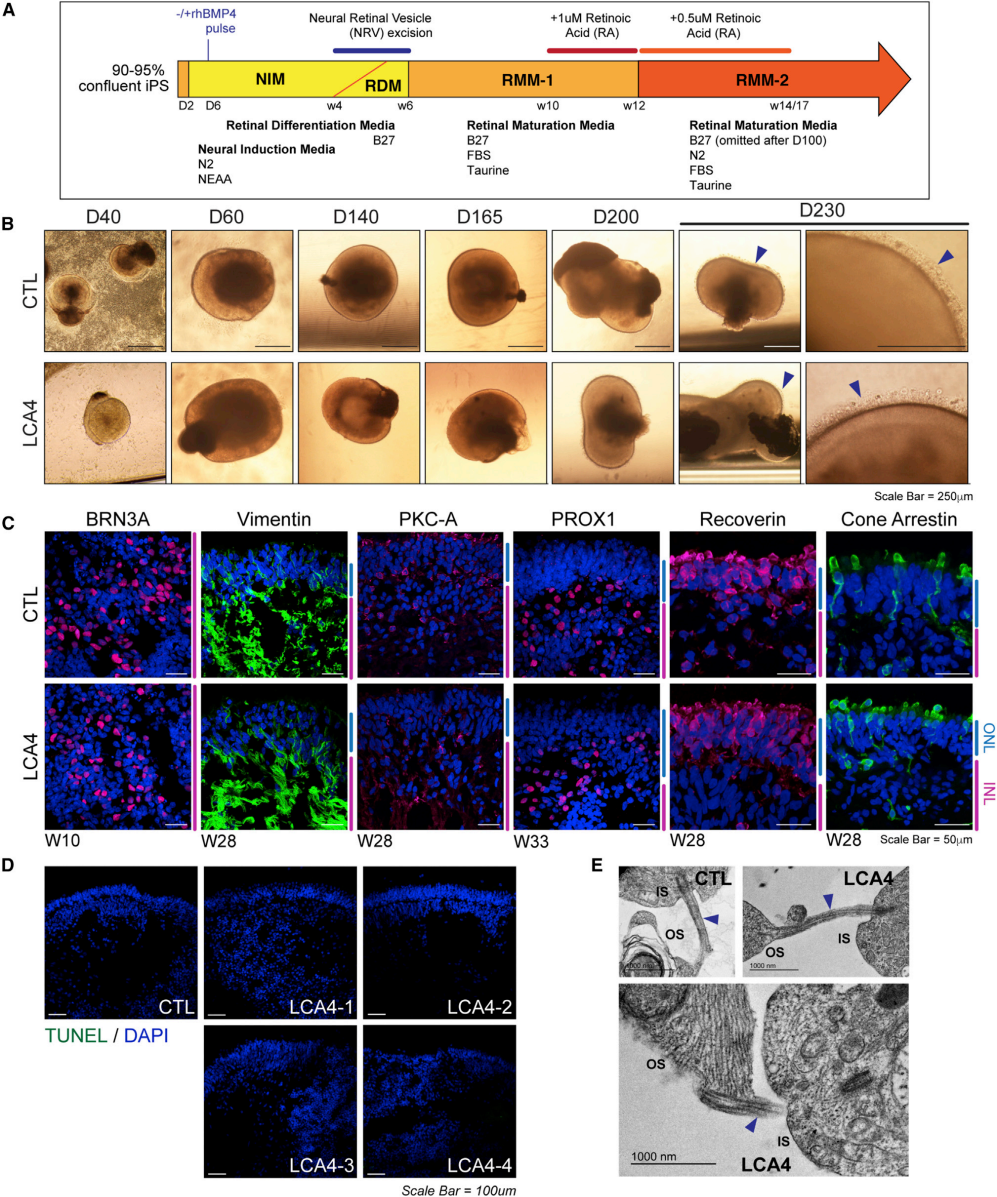
LCA4 iPSCs from RE cells were able to generate ROs with all retinal cell types (A) Schematic detailing the RO differentiation process. (B) Bright-field images of developing CTL and LCA4-1 ROs (days 40–230). A well-developed brush border of presumptive PR OSs/inner segments (ISs) was present on the surface of ROs by day 230 (blue arrowheads). Scale bars, 250 μm. Bright-field images of RO differentiation for LCA4-2, LCA4-3, and LCA4-4 are shown in Figure S3A. (C) IF images of CTL and LCA4 ROs for retinal cell markers: RGCs (BRN3A), Müller glia (vimentin), bipolar cells (PKC-A), amacrine/horizontal cells (PROX1), rod and cone PRs (recoverin), and cone PRs (cone arrestin). LCA4 images are representative of LCA4 ROs from all LCA4 lines. DAPI staining is blue. ONL and INL regions are highlighted at the side of images in blue and magenta. Scale bars, 50 μm. (D) TUNEL assay of week 28 CTL and LCA4 ROs. Very few TUNEL-positive cells (green) were observed. DAPI staining is blue. Scale bars, 100 μm. (E) TEM images of CTL and LCA4-1 RO PRs. Connecting cilia (blue arrowheads) were visible in both types of PRs. Outer segment (OS) structures contain membranous folds, and mitochondria were visible in ISs.

ROs were derived from iPSCs as described previously (Gonzalez-Cordero et al., 2017; Figure 2A). Clearly defined, neuro-retinal vesicles (NRVs) emerged after 3–4 weeks and displayed laminated photoreflective properties. Over time, the mechanically isolated NRVs expanded in size while maintaining a clearly laminated structure (the outer nuclear layer [ONL]) comprised of maturing photoreceptor (PR) cells. 24–26 weeks after NRV isolation, the ONL PR cells formed clear ciliary extensions and distal structures comprising a dense brush border of presumptive PR inner and outer segments (Figures 2B and S3A).

Gene expression analysis of week 8–33 CTL and LCA4 ROs for genes involved in early retinal development (*PAX6* and *VSX2*) and PR development (*CRX* and *NRL*) and function (recoverin [*RCVRN*], rhodopsin [*RHO*], cone opsins [*OPN1SW* and *OPN1MW/LW*], and phosphodiesterase subunits [*PDE6A*, *PDE6B*, *PDE6G*, *PDE6C*, and *PDE6H*]) indicated that the genes were regulated in a similar temporal pattern in CTL and LCA4 ROs (Figure S3B). Later onset of expression of PR-specific rod and cone phototransduction components (*OPN1SW*, *OPN1MW/LW*, *RHO*, *PDE6A*, *PDE6B*, and *PDE6G*) was observed. *AIPL1* expression was first detected at weeks 8–10 in CTL and LCA4 ROs with a noticeable increase at weeks 24–33, corresponding with increased expression of retinal PR markers. Similar patterns in CTL and LCA4 ROs were also observed for genes associated with non-PR retinal cell types, including retinal ganglion cells (RGCs) (*BRN3B* and *ISL1*), Müller glia (*CRALBP*), amacrine and horizontal cells (*PROX1*), and bipolar cells (*PKC-A*) (Figure S3B).

Immunofluorescence (IF) analysis of CTL and LCA4 ROs confirmed that the differentiation of mature LCA4 ROs from RE cell iPSCs derived from affected individuals was comparable with CTL ROs (Figure 2C). RGCs (BRN3A) were abundant throughout the core of early-staged CTL and LCA4 ROs. In later-staged, mature ROs (weeks 28–33), Müller glia (vimentin), bipolar cells (PKC-A), horizontal and amacrine cells (PROX1), and PRs (RCVRN [rods and cones] and CARR [cone arrestin]) were present in CTL and LCA4 organoids. TUNEL assays were conducted to assess cell death in the developing ROs (Figure 2D). Very few TUNEL-positive cells were detected in CTL and LCA4 ROs. Transmission electron microscopy (TEM) highlighted that the PRs in LCA4 ROs are structurally similar to those in CTL ROs, with clearly defined connecting cilia linking presumptive inner and outer segments (Figure 2E). These data indicate that development of the RO retinal tissue structure and cell types is comparable in CTL ROs and ROs from affected individuals and that overt indictors of neurodegeneration are absent in developing ROs from affected individuals.

### LCA4 ROs lack detectable AIPL1 protein despite the presence of *AIPL1* mRNA transcripts

**Figure 3:**
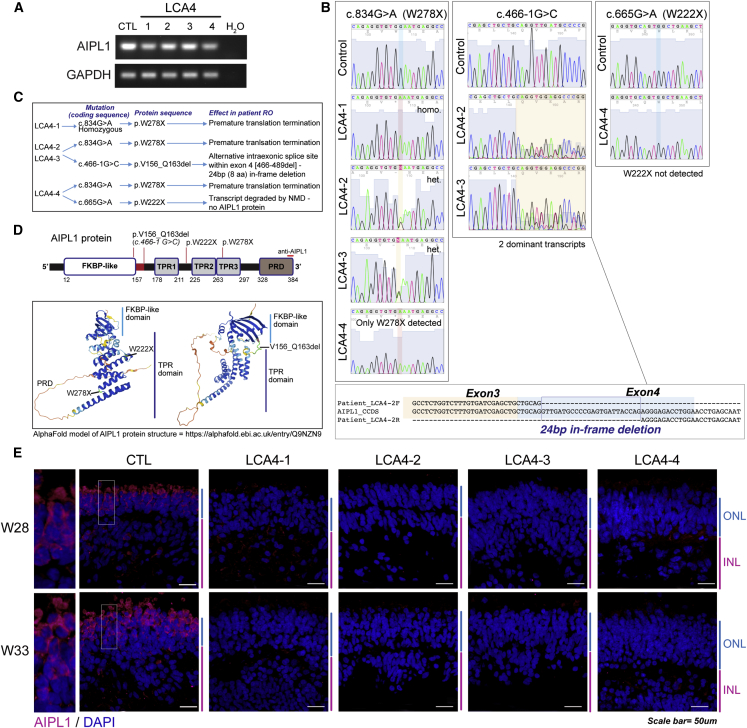
Analysis of AIPL1 transcript and protein in LCA4 ROs (A) Semi-quantitative PCR of the AIPL1 transcript present in all LCA4 ROs. (B) Amplification and Sanger sequencing of regions containing the c.834G>A, p.W278X; c.466-1G>C and c.665G>A, p.W222X mutations from LCA4 cDNA. (C) Summary of the effect of the LCA4-1, LCA4-2, LCA4-3, and LCA4-4 mutations on the AIPL1 transcript and protein. (D) Schematics of the AIPL1 2D and 3D protein structure. The location of the epitope targeted by the human-specific anti-AIPL1 C-terminal antibody is shown in the linear structure. The LCA4 mutation sites are highlighted. The 3D structure of AIPL1 is an AlphaFold model (AlphaFold: https://alphafold.ebi.ac.uk/entry/Q9NZN9). (E) IF analysis of AIPL1 in CTL and LCA4 ROs (week 28, week 33) with the C-terminal human-specific AIPL1 antibody. In CTL ROs, AIPL1 was present in the cell body and IS of the PRs located in the ONL (magnification). DAPI staining is blue. ONL and INL regions are highlighted at the side of images in blue and magenta. Scale bars, 50 μm.

ROs from all lines from affected individuals express *AIPL1* mRNA, as shown by PCR amplification of *AIPL1* (exon 1–2 region) from week 28 RO cDNA samples (Figure 3A). To determine the effect of the LCA4 mutations on *AIPL1* mRNA transcripts, the regions of interest (exons 3–5 for c.466-1G>C and c.665G>A, p.W222X and exons 5 and 6 for c.834G>A, p.W278X) were amplified from week 28 RO cDNA and sequenced (Figure 3B). The c.834G>A, p.W278X transcript was detected in all LCA4 ROs, indicating that this transcript does not undergo or partially escapes nonsense-mediated decay (NMD). The p.W278X nonsense mutation codes for a premature stop codon expected to induce C-terminal truncation of the AIPL1 TPR domain (Figures 3C and 3D). Similarly, the p.W222X nonsense mutation is expected to lead to AIPL1 C-terminal truncation (Figures 3C and 3D), but the c.665G>A, p.W222X transcript was not detected in LCA4-4 RO cDNA, suggesting that the mRNA transcript undergoes NMD, resulting in the absence of this protein in ROs from affected individuals (Figure 3B). Sequencing of LCA4-2 and LCA4-3 samples revealed that the c.466-1G>C mutation results in a transcript missing the first 24 bp of exon 4 (an in-frame, 8-amino-acid deletion; p.V156_Q163del) (Figure 3B). The in-frame p.V156_Q163del mutation leads to deletion of the linker between the FKBP-like and TPR domains (Figures 3C and 3D).

IF was carried out on week 28 and week 33 LCA4 ROs with an AIPL1 polyclonal antibody targeted to the human-specific C terminus (van der Spuy et al., 2002; Figure 3E). AIPL1 protein was specifically detected in the PRs of CTL iPSC ROs. In contrast, AIPL1 protein was not detected at any time point in the LCA4 ROs (Figure 3E). Because the p.W278X mutation leads to premature translation termination and C-terminal truncation of AIPL1, the missing antibody epitope may account for the lack of detectable p.W278X expression in LCA4 ROs. However, the lack of detectable AIPL1 levels in all LCA4 ROs was confirmed with a second well-characterized anti-AIPL1 antibody raised against recombinant purified full-length human AIPL1 (Figure S3C; Ramamurthy et al., 2003). Therefore, these results suggest that the p.W278X and p.V156_Q163del proteins, whose respective transcripts were expressed in the ROs from affected individuals, likely misfold and may be rapidly degraded, whereas the p.W222X product is not detected as a result of NMD of the transcript.

### ROs derived from affected individuals recapitulate the key molecular features of LCA4 *in vitro*

**Figure 4:**
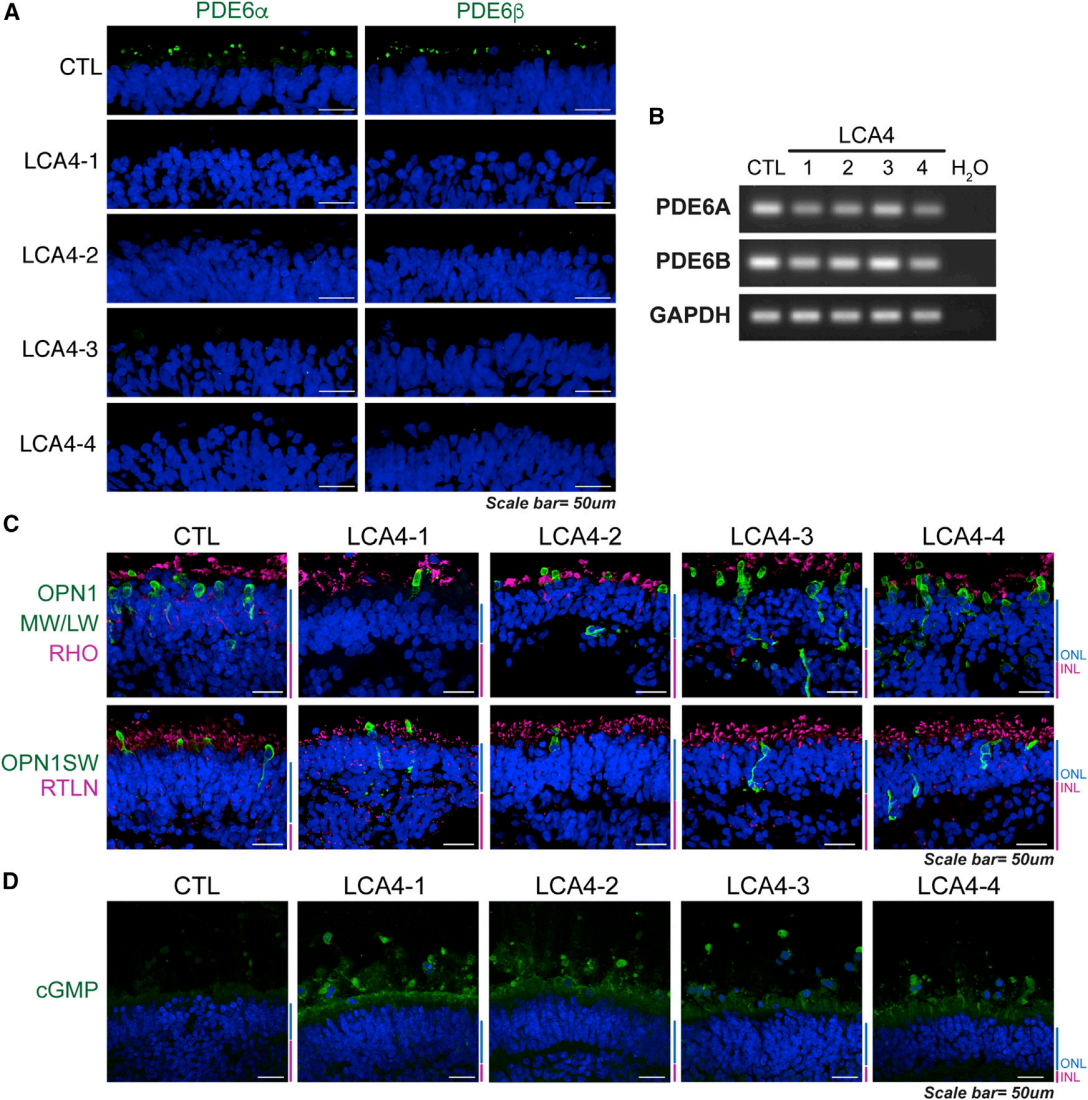
PRs in LCA4 ROs lacked detectable expression of rod cGMP PDE6α/PDE6β and displayed elevated levels of cGMP (A) IF analysis of rod cGMP PDE6α and PDE6β proteins. DAPI staining is blue. Scale bars, 50 μm. (B) Semi-quantitative PCR of *PDE6A* and *PDE6B* with the housekeeping gene GAPDH. *PDE6A* and *PDE6B* transcripts were present in all LCA4 ROs. (C) IF analysis of week 33 RO sections for opsins (OPN1MW/LW, OPN1SW), rhodopsin (RHO), and rootletin (RTLN). DAPI staining is blue. ONL and INL regions are highlighted at the side of images in blue and magenta. Scale bars, 50 μm. (D) IF analysis of cGMP in week 28 ROs. DAPI staining is blue. ONL and INL regions are highlighted at the side of images in blue and magenta. Scale bars, 50 μm.

Post-transcriptional loss of PDE6 subunits has been reported in AIPL1 loss-of-function animal models (Iribarne et al., 2017; Kirschman et al., 2010; Kolandaivelu et al., 2014; Liu et al., 2004; Ramamurthy et al., 2004). Similarly, transcriptomics analysis of human LCA4 ROs confirmed post-transcriptional loss of PDE6 (Lukovic et al., 2020). IF analysis of week 28 CTL and LCA4 ROs revealed that, although PDE6α and PDE6β were localized to the presumptive rod PR outer segments in the CTL ROs, both subunits were completely absent in the LCA4 RO PRs (Figure 4A). *PDE6A* and *PDE6B* transcripts were detected in all week 28 RO samples (Figure 4B), confirming post-transcriptional loss of PDE6. Rhodopsin (RHO) and cone opsin (OPN1LW/MW and OPN1SW) localization showed that the pattern of distribution and morphology of the PR cell populations do not differ in LCA4 ROs compared with CTL ROs (Figure 4C).

The PDE6 complexes play a crucial role in cGMP hydrolysis. To ascertain whether cGMP levels were raised in LCA4 ROs compared with CTL, week 28 ROs were immunostained for cGMP. CTL ROs exhibited low background detection of cGMP. In contrast, increased cGMP was detected in all 4 LCA4 ROs from affected individuals Figure 4D).

### PTC124 treatment rescued partial levels of full-length AIPL1 in LCA4 ROs homozygous for p.W278X

**Figure 5:**
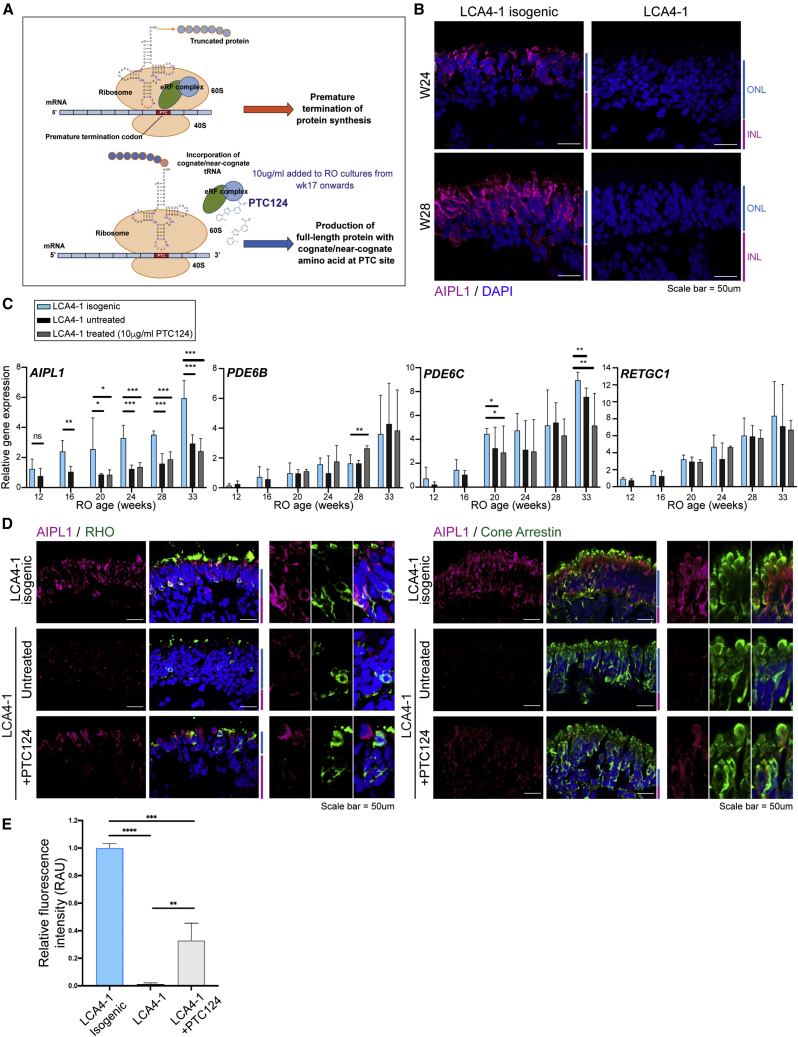
CRISPR-Cas9 repair of p.W278X in LCA4-1 iPSCs restored AIPL1 protein levels, and PTC124 treatment was able to partially rescue AIPL1 protein levels in LCA4-1 ROs (A) Schematic detailing the proposed mechanism of action of PTC124 in driving translation readthrough of premature termination codons (PTCs). (B) IF analysis of LCA4-1 isogenic ROs (week 24, week 28) demonstrated restoration of AIPL1 protein to the ONL PR cells. DAPI staining is blue. ONL and INL regions are highlighted at the side of images in blue and magenta. Scale bars, 50 μm. (C) qPCR analysis of *AIPL1*, *PDE6B*, *PDE6C*, and *RETGC1* levels in LCA4-1 isogenic and LCA4-1 untreated and treated (10 μg/mL PTC124) ROs (weeks 12–33). Gene expression levels were normalized to the PR marker *CRX*. Levels of *CRX* normalized to β-actin are shown in Figure S6B. Representative results are shown for qPCR analysis of 3 biological replicates (individual ROs) per time point; a minimum of two differentiations were conducted for each line. Graphs show mean ± SD; ^∗^p ≤ 0.05, ^∗∗^p ≤ 0.01. (D) IF analysis of ROs for AIPL1, RHO, and CARR expression in untreated and PTC124-treated ROs. DAPI staining is blue. ONL and INL regions are highlighted at the side of images in blue and magenta. Scale bars, 50 μm. (E) Fluorescence intensity measurements for AIPL1 immunoreactivity in week 24 RO ONL regions. The relative fluorescence intensity (ImageJ) was calculated for 3–5 images per RO type. Graphs show mean ± SD; the significance level (2-tailed t test) is denoted as follows: ^∗∗^p ≤ 0.01, ^∗∗∗^p ≤ 0.001, ^∗∗∗∗^p ≤ 0.0001.

Our data confirm that the c.834G>A, p.W278X transcript largely escapes NMD and is therefore a potentially suitable target for TRID therapy. PTC124 facilitates incorporation of cognate/near-cognate amino acids at PTC sites via a mechanism involving inhibition of release factor (eRF1/eRF3) activity at the ribosomal complex (Figure 5A; Ng et al., 2021). PTC124 was added to RO cultures from week 17 onward. A pilot study short-term (2-week) PTC124 dosage gradient (5–15 μg/mL) revealed that PTC124 could induce readthrough of full-length AIPL1, with 10–12.5 μg/mL being most efficient at driving p.W278X readthrough and increased readthrough not observed with increased PTC124 doses (Figure S4). PTC124 was thus used at a concentration of 10 μg/mL in all experiments.

To generate optimal CTLs for investigation of the effect of PTC124 on LCA4-1 ROs, isogenic repair lines were generated via CRISPR-Cas9 homology-directed repair (HDR) (Figure S5A; Table S4). Two isogenic LCA4-1 iPSC lines (homozygous repair of the p.W278X locus) were further characterized with regard to expression of pluripotency markers and trilineage differentiation potential (Figures S5B–S5D). No off-target editing was observed in the isogenic lines at the top 10 predicted off-target genomic loci (Table S5). In ROs derived from the LCA4-1 isogenic iPSCs, AIPL1 was detected in PR cells as expected. CRISPR-Cas9 HDR therefore restored AIPL1 protein expression (Figure 5B).

Gene expression analysis of week 12–33 CTL isogenic and LCA4-1 ROs (untreated/treated) for genes involved in early retinal development (*VSX2*), PR function (*RCVN*, *RHO*, *OPN1SW*, and *OPN1MW/LW*), and non-PR retinal markers (*PKC-A*, *PROX1*, and *CRALBP*) indicated that these genes were regulated in a similar temporal pattern in the isogenic CTL and LCA4-1 ROs (untreated/treated) (Figure S6A). Quantitative gene expression analysis of isogenic and LCA4-1 (untreated/treated) ROs surprisingly revealed that LCA4-1 ROs consistently express approximately half the amount of AIPL1 transcript compared with the isogenic ROs (Figure 5C). This was an unexpected finding, given that we detected the *AIPL1* p.W278X transcript in all ROs and that it is not expected to undergo NMD. Levels of the transcript were not changed in PTC124-treated ROs compared with untreated ones. In contrast to the changes in *AIPL1* levels, expression levels of rod *PDE6B*, cone *PDE6C* and *RETGC1* were comparatively similar in the isogenic and LCA4 ROs, and PTC124 treatment did not significantly affect the transcript levels of *AIPL1*, *PDE6B*, *PDE6C* or *RETGC1* (Figure 5C). Transcript levels of the PR marker *CRX* were also comparable between all sample types (Figure S6B).

IF staining was carried out on untreated and PTC124-treated LCA4-1 ROs with the AIPL1 C-terminal antibody to detect readthrough of full-length AIPL1 from the p.W278X transcript (Figure 5D). RHO and CARR colocalized with subsets of AIPL1-positive cells in the LCA4-1 isogenic ROs, demonstrating that AIPL1 is present in rod- and cone-lineage PRs (Figure 5D, week 24 RO). In LCA4-1 ROs treated with 10 μg/mL PTC124, AIPL1 immunoreactivity was significantly elevated above untreated ROs and reached ∼32.8% ± 12.6% compared with the isogenic CTL (Figure 5E). RHO+ AIPL1+ and CARR+ AIPL1+ cells were present in the treated ROs (Figure 5D, week 24 RO); PTC124 is therefore able to promote readthrough of the AIPL1 PTC in both types of PR cells. PR markers (transducin, OPN1SW, and RHO) were similar in LCA4-1 isogenic and LCA4-1-untreated and -treated ROs (Figure S6C), and the level of cell death (TUNEL assay) was not increased in LCA4-1 ROs or in the presence of 10 μg/mL PTC124 (Figure S6D). Therefore, long-term dosage with PTC124, although promoting readthrough of full-length AIPL1 protein, did not have deleterious, toxic effects on RO cell survival.

### PTC124 treatment showed limited rescue of rod PDE6 in p.W278X homozygous LCA4 ROs

**Figure 6:**
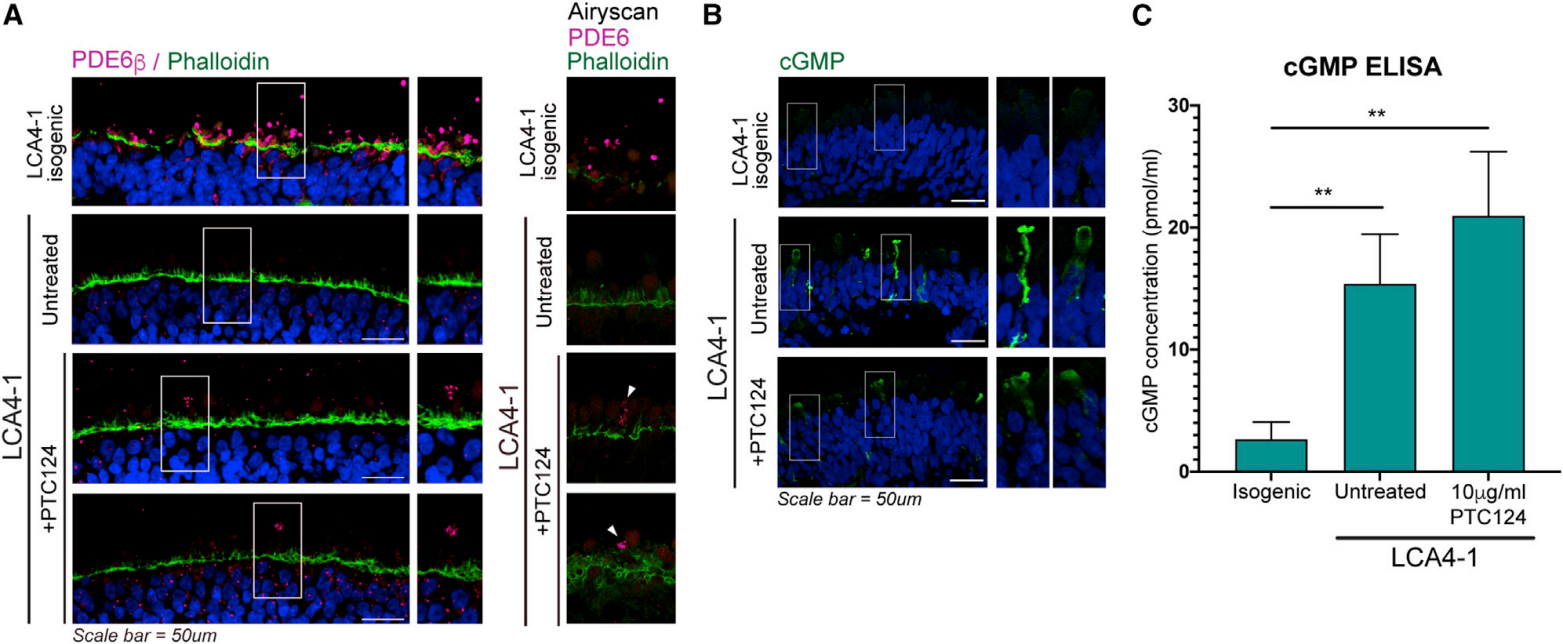
PTC124 treatment was able to restore PDE6β in a limited number of PRs in LCA4-1 ROs but had no effect on cGMP levels (A) IF analysis of ROs (week 28) for PDE6β. Rescue of PDE6β was observed in the IS/OS region of a small number of PRs in PTC124-treated LCA4-1 ROs (white arrowheads, Airyscan images). DAPI staining is blue. Scale bars, 50 μm. (B) IF analysis (week 28) showed that cGMP was not detected in LCA4-1 isogenically repaired ROs. cGMP-positive PR cells were present in LCA4-1 untreated and PTC124-treated ROs. DAPI staining is blue. Scale bars, 50 μm. (C) cGMP ELISA analysis of whole ROs (week 33). 3–5 biological replicates (individual ROs) of each sample (isogenic, untreated, treated) from the same differentiation were analyzed, with the experiment repeated twice (minimum of two differentiations per line). The graph shows mean ± SD. ^∗∗^p ≤ 0.01.

Because PTC124 was able to rescue low levels of full-length AIPL1 in LCA4-1 ROs, we investigated the recovery of rod PDE6β and cGMP levels. Isogenic repair of c.834G>A, p.W278X in LCA4-1 ROs not only restored the expression and correct localization of AIPL1 but also that of PDE6β in the presumptive rod PR outer segments (Figure 6A). LCA4-1 PTC124-treated ROs displayed rescue of PDE6β in a small subset of PRs compared with the isogenic CTL, with the protein correctly localized to the presumptive outer segment (OS) region of the cells (Figure 6A).

Similar to CTL ROs, cGMP levels were undetectable in PR cells in LCA4-1 isogenic ROs (Figure 6B). In comparison, a number of cGMP + PRs were visible in LCA4-1 ROs. PTC124 treatment, however, had no discernible effect on cGMP (Figure 6B). This finding was confirmed by cGMP ELISA results with whole-organoid material, which revealed significant elevation of cGMP in the LCA4-1 ROs from affected individuals compared with the isogenic CTL. However, there was no significant change in cGMP levels in PTC124-treated compared with untreated ROs (Figure 6C). Therefore, the partial readthrough of low levels of full-length AIPL1 and restoration of PDE6 in a limited number of PRs was not sufficient to reduce cGMP levels in whole organoids.

### PTC124 induced low levels of readthrough in LCA4 ROs compound heterozygous for p.W278X

**Figure 7:**
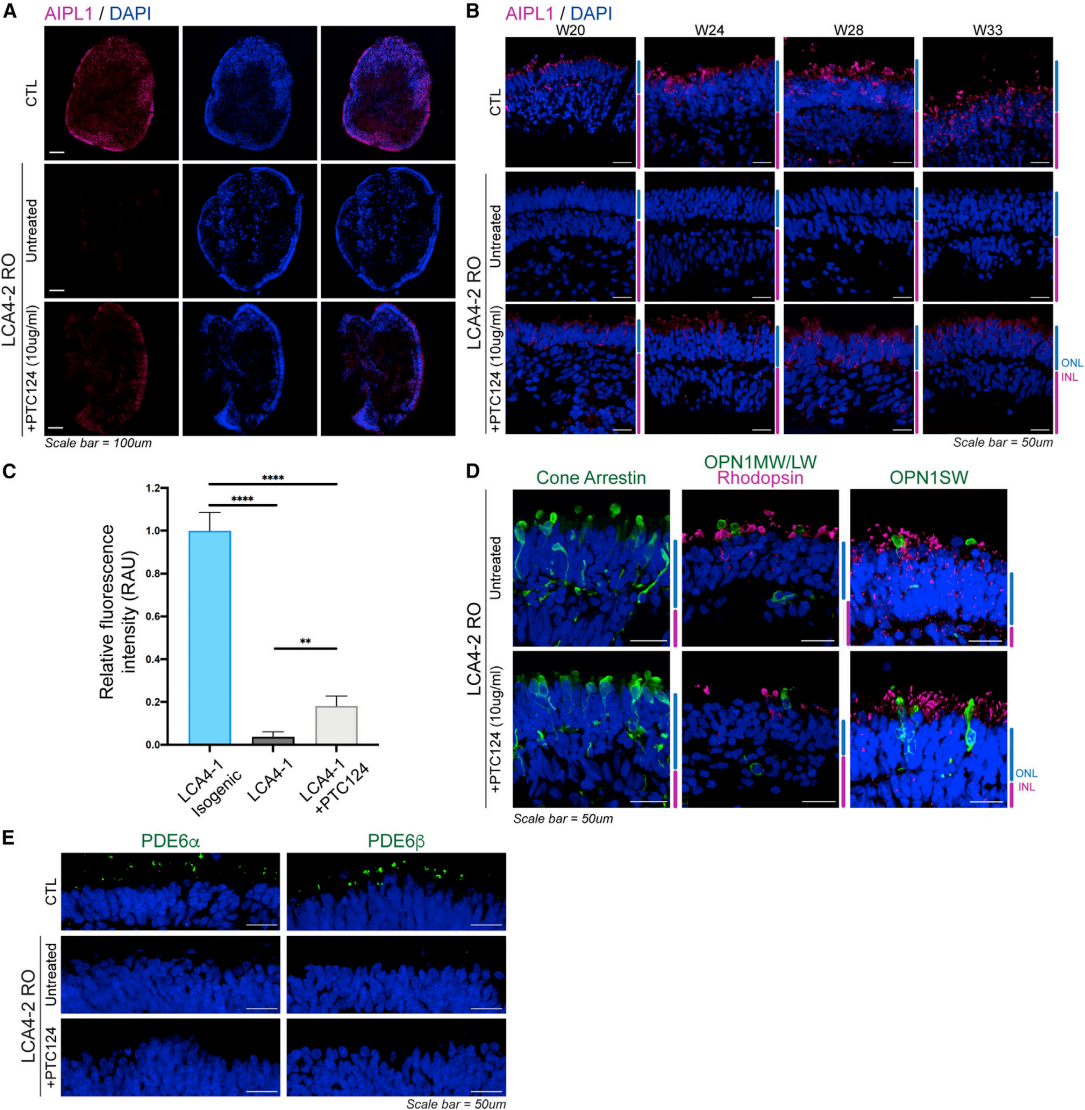
PTC124 translation readthrough treatment was able to rescue low levels of AIPL1 in LCA4 compound heterozygous ROs (A) AIPL1 IF of week 28 CTL, LCA4-2 untreated, and LCA4-2 treated (10 μg/mL PTC124) ROs, shown in whole organoids. DAPI staining is blue. Scale bars, 100 μm. (B) IF analysis of AIPL1 levels in week 20–33 CTL and LCA4-2 ROs (untreated/treated with 10 μg/mL PTC124). DAPI staining is blue. ONL and INL regions are highlighted at the side of images in blue and magenta. Scale bars, 50 μm. (C) Fluorescence intensity measurements (ImageJ) of AIPL1 immunoreactivity in week 24 RO ONL regions, calculated for 3–5 images per RO type. Graphs show mean ± SD. The significance level (2-tailed t test) is denoted as follows: ^∗∗^p ≤ 0.01, ^∗∗∗∗^p ≤ 0.0001. (D) IF for CARR, OPN1MW/LW, RHO, OPN1SW, and ciliary RTLN in LCA4-2 untreated/treated ROs. DAPI staining is blue. ONL and INL regions are highlighted at the side of images in blue and magenta. Scale bars, 50 μm. (E) IF analysis of PDE6α and PDE6β in CTL, untreated, and PTC124-treated LCA4-2 ROs (week 28). DAPI staining is blue. Scale bars, 50 μm.

Because the c.834G>A, p.W278X mutation is prevalent in individuals with LCA4 compound heterozygous for this allele, we also tested PTC124-mediated readthrough in LCA4-2 ROs that are compound heterozygous for c.834G>A, p.W278X and the c.466-1G>C splice mutation. ROs between weeks 20 and 33 were collected to study the effect of PTC124 treatment on AIPL1 protein levels. IF with the AIPL1 C-terminal antibody indicated that there was rescue of full-length AIPL1 at all time points studied (Figure 7A, 10× magnification images [week 28 RO]; Figure 7B, 40× of the ONL region [week 20–33 Ros]). IF analysis (week 24 ROs) indicated that the rescue induced by PTC124 was significant compared with LCA4-2 untreated ROs and reached ∼18.1% ± 4.7% of levels seen in CTL ROs (Figure 7C). PTC124 treatment itself had no obvious effect on the morphology of retinal cell types, including rods and cones, or retinal tissue layers (Figure 7D). Because PTC124 was able to rescue low levels of AIPL1 protein in treated LCA4-2 ROs, IF was conducted to ascertain whether this was sufficient to rescue rod PDE6 in LCA4-2 ROs. Analysis of week 28 sections for PDE6α and PDE6β protein demonstrated that, in contrast to LCA4-1 ROs homozygous for p.W278X, there was no observable elevation of PDE6 protein in PTC124-treated p.W278X heterozygous LCA4-2 ROs (Figure 7E).

## Discussion

In this study, we developed and characterized the first human AIPL1-LCA4 iPSC RO model derived from RE cells and developed an isogenic CTL through homozygous repair of the c.834G>A, p.W278X mutation in the LCA4-1 line. This *in vitro* model is a powerful tool to understand different mechanisms of pathogenesis of *AIPL1* variations. We identified that transcripts from the c.466-1G>C splice variation and c.834G>A, p.W278X nonsense variation result in AIPL1 protein species that may undergo rapid degradation. In contrast, the AIPL1 c.665G>A, W222X nonsense variation involves production of a transcript that undergoes NMD.

We report complete loss of AIPL1 protein, loss of rod cGMP PDE6α and PDE6β subunits, and elevation of cGMP levels in ROs from four individuals with LCA4 sharing 3 different *AIPL1* genotypes. We show that the LCA4 ROs follow the spatiotemporal development of the retina, with no overt indicators of PR degeneration in the developing ROs. These findings are in agreement with a recently published human RO model of LCA4 harboring the homozygous AIPL1 mutation p.C89R (Lukovic et al., 2020). In *Aipl1* knockout mice, where PR degeneration begins at post-natal day 9 (P9) and is complete by P30, the PR presynaptic terminals develop normally (Singh et al., 2014). However, defective expression of postsynaptic proteins in bipolar cells is noted prior to onset of PR degeneration in the developing retina at P8. Significant inner retinal remodeling proceeds after onset of PR degeneration from P14 onward, coincident with PR degeneration. Similar changes are likely to occur in individuals with LCA4 prior to and after onset of PR degeneration.

Individual-specific ROs are a powerful tool to test new therapeutic agents for IRDs. Our data show that treatment of ROs with PTC124 restored AIPL1 levels to ∼33 and ∼18% of CTL levels in p.W278X homozygous and heterozygous ROs, respectively. The optimal dose of PTC124 in our study was 10–12.5 μg/mL, and we showed no retinal toxicity at this concentration. Excellent retinal biocompatibility at an effective concentration of 10 μg/mL has been reported in nonsense-mediated IRDs, with no retinal cytotoxicity observed at any dose tested (Goldmann et al., 2011, 2012; Schwarz et al., 2015; Ramsden et al., 2017; Samanta et al., 2019; Liu et al., 2020; Vössing et al., 2020). Accordingly, PTC124 systemic dosing regimens in animal models (Welch et al., 2007; Du et al., 2008) and human clinical trials (Hirawat et al., 2007; Finkel et al., 2013; Bushby et al., 2014) have been designed to maintain target plasma concentrations of at least 2–10 μg/mL. These studies have also shown PTC124 to be safe and effective at these concentrations with no adverse effects. Increasing the concentration of PTC124 in our RO model did not further enhance the readthrough level, indicating a threshold for readthrough efficiency in our system. Similarly, an inverted bell-shaped activity-response curve has been reported in zebrafish and mouse models of DMD (Welch et al., 2007; Li et al., 2014) and in individuals with DMD (Finkel et al., 2013; Bushby et al., 2014) as well as a zebrafish model of retinal choroideremia (Moosajee et al., 2016). The increased level of full-length AIPL1 protein mediated by PTC124 readthrough was, however, not sufficient to restore rod PDE6 to the levels required to reduce cGMP.

The efficiency of readthrough to restore native protein function is dependent on the amount of target transcript, the nucleotide context of the PTC, and the features of the full-length protein arising from the readthrough event. The efficiency of phenotypic rescue was greater in ROs homozygous for c.834G>A, p.W278X, which could be explained by the increased amount of target transcript available for readthrough. The c.834G>A, p.W278X mutation is located 50 nt downstream of the final exon 5-exon 6 junction, and the transcript is thus expected to be resistant to NMD (Hug et al., 2016). Indeed, the AIPL1 c.834G>A, p.W278X transcript was detected in all ROs derived from affected individuals.

PTC124 selectively induces readthrough of PTC over natural stop codons and promotes translational readthrough of all three stop codons, with the highest efficiency for UGA, followed by UAG and UAA (Manuvakhova et al., 2000; Welch et al., 2007). The p.W278X termination codon is UGA and, therefore, expected to yield the highest levels of readthrough. The PTC124 readthrough efficiency is also influenced by the nucleotide immediately 3′ to the termination codon, where increased efficiency is favored by a pyrimidine base, especially cytosine (Manuvakhova et al., 2000; Welch et al., 2007). This position is occupied by adenine at the c.834G>A, p.W278X locus, which may reduce readthrough efficiency. Insertion of a near-cognate tRNA coding for tryptophan at the UGA PTC, which would reinstate the AIPL1 wild-type sequence, is only one of several possibilities. Characterization of translational readthrough products from PTC124-treated 293H cells revealed predominant insertion of arginine (∼69%), followed by tryptophan (∼28%) and cysteine (∼0.7%), at the UGA PTC (Roy et al., 2016). Therefore, less than a third of the rescued AIPL1 recovered in PTC124-treated ROs may be wild-type AIPL1 harboring a reinstated tryptophan residue. *In silico* predictions of p.W278R and p.W278C predict that insertion of arginine or cysteine is neutral or probably deleterious, respectively (Rhapsody) (Ponzoni et al., 2020), or that both are deleterious (PolyPhen2). The W278 residue occupies an important structural position in the AIPL1 TPR domain, and the substitution of tryptophan with arginine or cysteine is likely to be poorly tolerated at this position. Our data suggest that low levels of readthrough in our ROs combined with heterogeneity in the incorporation of the near-cognate amino acid could explain the sub-therapeutic rescue and amelioration of the disease phenotype. It has been shown that PTC124 potentiated G418-stimulated readthrough, suggesting additivity of the combined action of these TRIDs (Ng et al., 2021). Therefore, the recovery of rod PDE6 subunits in a small number of PRs in our study suggests that combination therapies based on the orthogonal mechanisms of action of PTC124 and less toxic aminoglycoside derivatives could be beneficial to further increase AIPL1 p.W278X readthrough.

## Experimental procedures

### Ethical approval

To collect urine samples for iPSC generation, parents or legal guardians of children with LCA4 (who were all under the age of 3 years) signed an informed consent form in adherence with the Declaration of Helsinki and with approval from the North East – Newcastle & North Tyneside 2 Research Ethics Committee.

### Differentiation of ROs

Directed differentiation of iPSCs into 3D ROs was based on a protocol described previously (Gonzalez-Cordero et al., 2017).

### Translational readthrough treatment

The TRID ataluren (3-[5-(2-fluorophenyl)-1,2,4- oxadiazol-3-yl]-benzoic acid) (Translarna or PTC124) was purchased from Selleckchem. Translational readthrough treatment of ROs with PTC124 started at week 17. Fresh PTC124 was diluted in retinal maturation medium at a final concentration of 5–25 μg/mL and added to the ROs every other day when changing the medium.

### IF and imaging

ROs were fixed in 4% paraformaldehyde (PFA), 5% sucrose in PBS for 30 min at 4°C and dehydrated in 6.25%, 12.5%, and 25% sucrose:PBS (1-h incubations at 4°C, ROs left in 25% sucrose overnight). ROs were embedded in OCT (Tissue-Tek). 7-μm cryosections were mounted on Super-Frost Plus slides (Thermo Scientific). Slides were incubated in 10% donkey serum (Sigma-Aldrich) or fetal bovine serum (FBS) (Gibco), 0.01% Triton X-100 (Sigma-Aldrich) in PBS for 1 h at room temperature (RT) before a 1-h primary antibody incubation (Table S1). Slides were washed 3 times with PBS, incubated with 1:1,000 species-specific secondary antibody (Table S1) for 1 h, washed 3 times, and incubated with 4′,6-diamidino-2-phenylindole (DAPI; 2 mg/mL) (Invitrogen) in PBS for 5 min. Slides were mounted in fluorescence mounting medium (Dako). TUNEL staining was carried out on PFA-fixed sections using the *In Situ* Cell Death Detection Kit, Fluorescein (Roche) according to the manufacturer’s instructions. All images were acquired using LSM700 and LSM710 laser-scanning confocal microscopes (Carl Zeiss). Images were exported from Zen 2009 (Carl Zeiss) software and prepared using Adobe Photoshop, ImageJ (National Institutes of Health, Bethesda, MD, USA), and Adobe Illustrator CS6. For fluorescence intensity analysis (minimum of 3–5 measurements per treatment group), ImageJ was used to assess the fluorescence intensity of the ONL regions. Background threshold settings were identical for all measurements.

### RNA extraction and quantitative PCR (qPCR)

RNA from iPSC and ROs (minimum of 3 ROs for each time point and sample type) was extracted using the RNeasy Micro Kit (QIAGEN). cDNA synthesis was performed using the Tetro cDNA synthesis kit (Bioline). 2× GoTaq Green Master Mix (Promega) was used for DNA amplification by PCR with standard cycling conditions for semi-quantitative PCRs. Real-time PCR reactions were set up with 2× LabTaq Green Hi Rox Master Mix (Labtech) and validated primers (at a concentration of 0.25 pmol/μL) and run on an Applied Biosystems QuantStudio 6 Flex real-time PCR system. Primer sequences used for semi-quantitative/qPCR are detailed in Table S3. Gene expression levels were calculated using the ΔΔCt method; RO markers were normalized against CRX and β-actin. CRX was chosen as a consistently expressed PR-specific reference gene to negate differences in RO size/cellular make-up between samples.

### TEM

ROs were processed for TEM analysis and imaged as described previously (Lane et al., 2020).

### cGMP ELISA

96-well cGMP ELISA kits (Cayman Chemicals) were used according to the manufacturer’s instructions. ROs were washed with PBS and then incubated in 100 μL of 0.1 M HCl for 20 min at RT before mechanical homogenization. The samples were centrifuged at 1,000 × *g* for 10 min, and supernatants were collected. 200 μL of ELISA buffer was added to each sample (individual ROs; minimum of 3 ROs were processed per RO type). 50 μL was used per ELISA well (3 technical replicates per sample); samples were not acetylated for the analyses. Absorbance was measured at a wavelength of 420 nm.

### Statistical analysis

All lines, with the exception of LCA4-3 and LCA4-4, were differentiated to completion a minimum of twice. Biological replicates were obtained from and compared with different sample types from the same differentiation experiment. For real-time PCR analysis of gene expression levels and the cGMP ELISA analyses, group average and standard deviation (SD) were calculated from a minimum of 3 biological replicates (individual ROs) per sample/RO type. Pairwise comparisons were carried out using 2-tailed Student’s t tests (^∗^p ≤ 0.05, ^∗∗^p ≤ 0.01; annotated on the relevant graphs).

## Author contributions

A.L., A.S.-R., P.R.L.P., and H.S. performed the experiments and/or analyzed the data. M.G. and A.K. performed clinical studies and data analysis. A.-J.F.C., P.J.C., M.M., J.B., and M.E.C. provided materials, laboratory samples, or participants for the research. A.L., A.S.-R., and J.v.d.S. conceived the hypothesis, designed the experiments, and drafted the manuscript. All authors edited the draft manuscript.

## Supplemental information

Document S1. Figures S1–S6, Tables S1–S5, and supplemental experimental procedures

Document S2. Article plus supplemental information
