# Supplementary material for: Investigation of PTC124-mediated translational readthrough in a retinal organoid model of AIPL1-associated Leber congenital amaurosis
Source: Stem Cell Reports. 2022 Sep 8;17(10):2187–202. doi: 10.1016/j.stemcr.2022.08.005 (PMC9561542; doi:10.1016/j.stemcr.2022.08.005)
Supplement: Document S1. Figures S1–S6, Tables S1–S5, and supplemental experimental procedures [file mmc1.pdf]

Stem Cell Reports, Volume *17*

Supplemental Information

Investigation of PTC124-mediated translational readthrough in a retinal organoid model of AIPL1-associated Leber congenital amaurosis

Amy Leung, Almudena Sacristan-Reviriego, Pedro R.L. Perdigão, Hali Sai, Michalis Georgiou, Angelos Kalitzeos, Amanda-Jayne F. Carr, Peter J. Coffey, Michel Michaelides, James Bainbridge, Michael E. Cheetham, and Jacqueline van der Spuy

<u>Supplemental Information</u>

Supplemental Figures

Fig. S1: Molecular and clinical investigation of LCA4 patients, related to Fig. 1

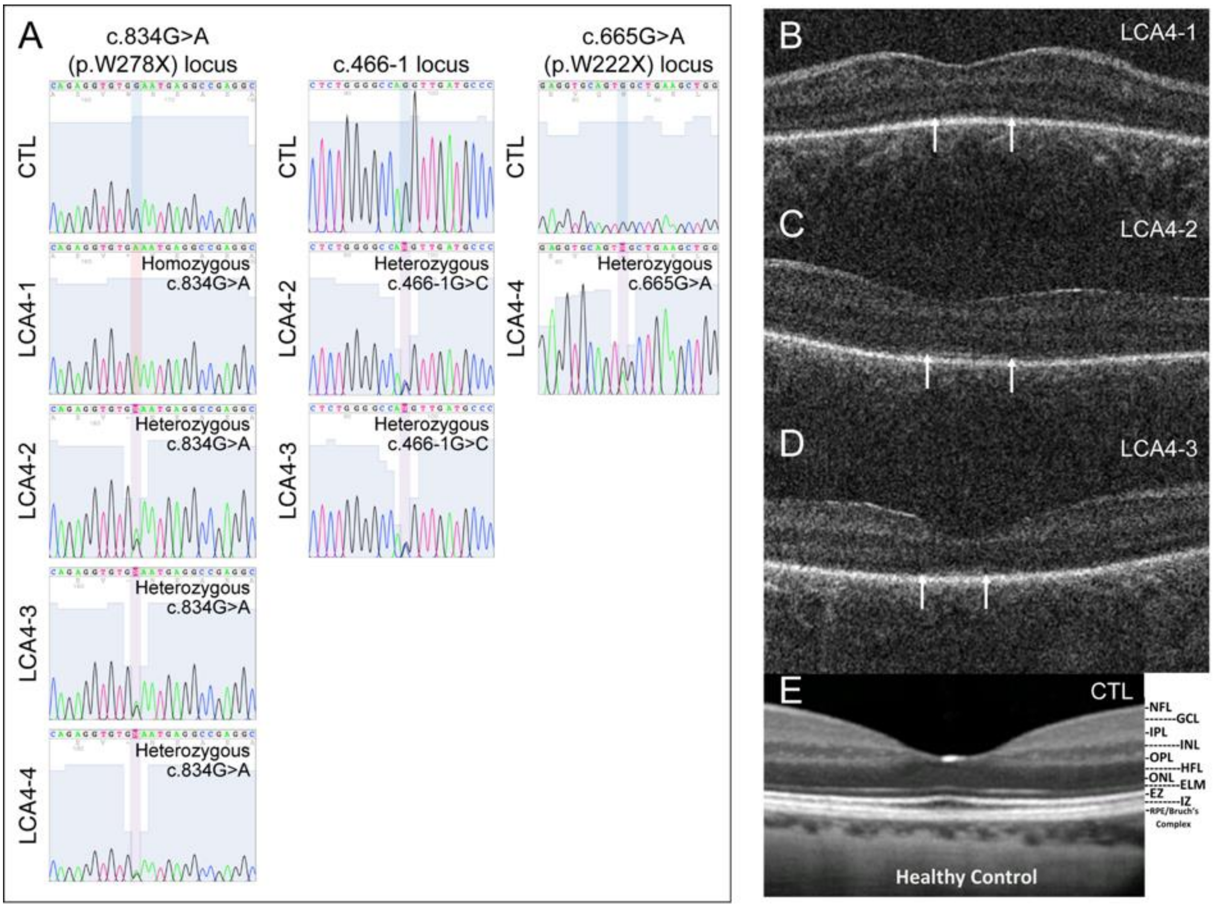

Fig. S2: Reprogramming of LCA4 patient renal epithelial cells and characterisation of iPSC lines, related to Fig. 1

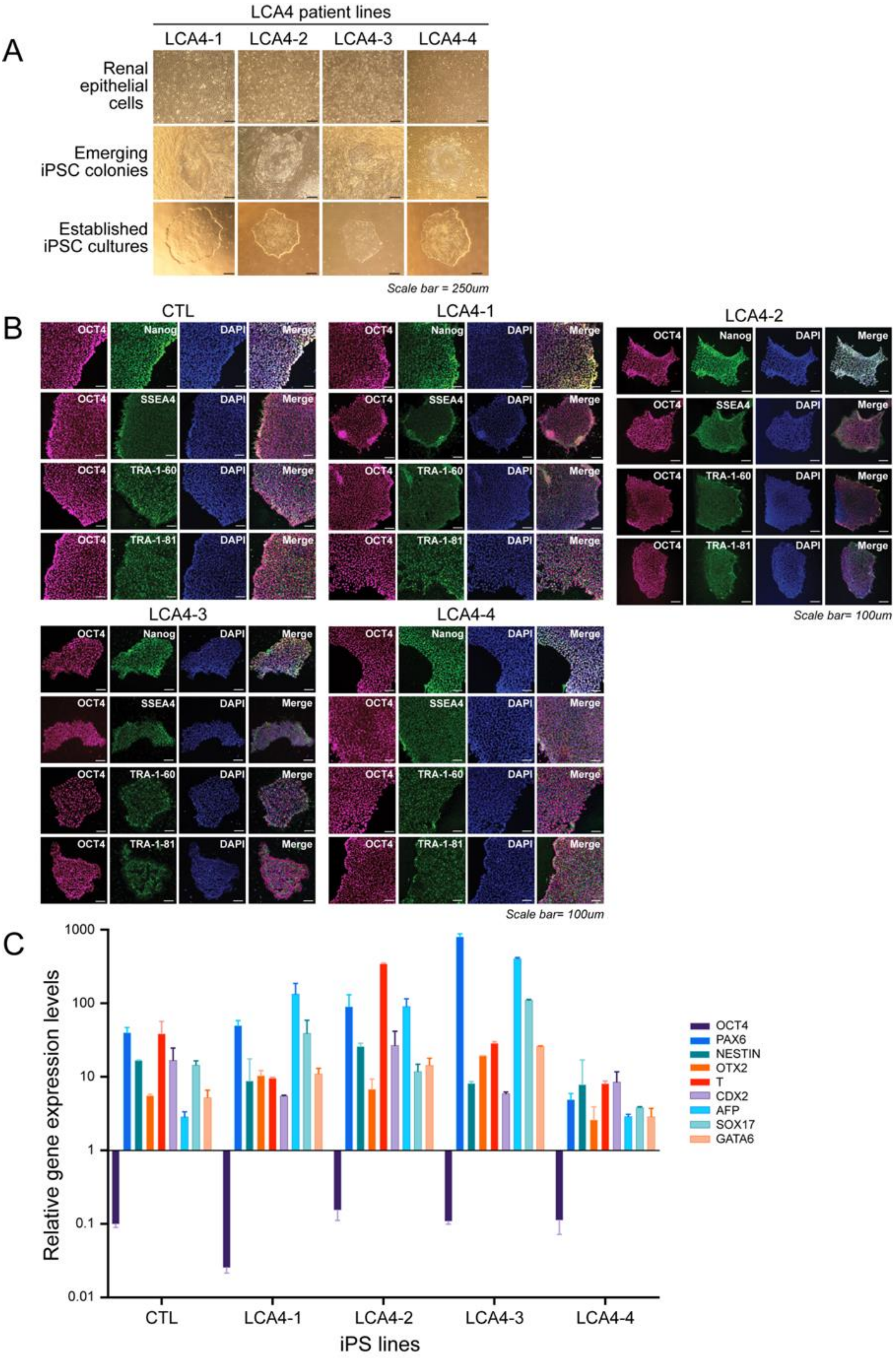

Fig. S3: Additional characterisation of LCA4 patient ROs, related to Fig. 2 and 3

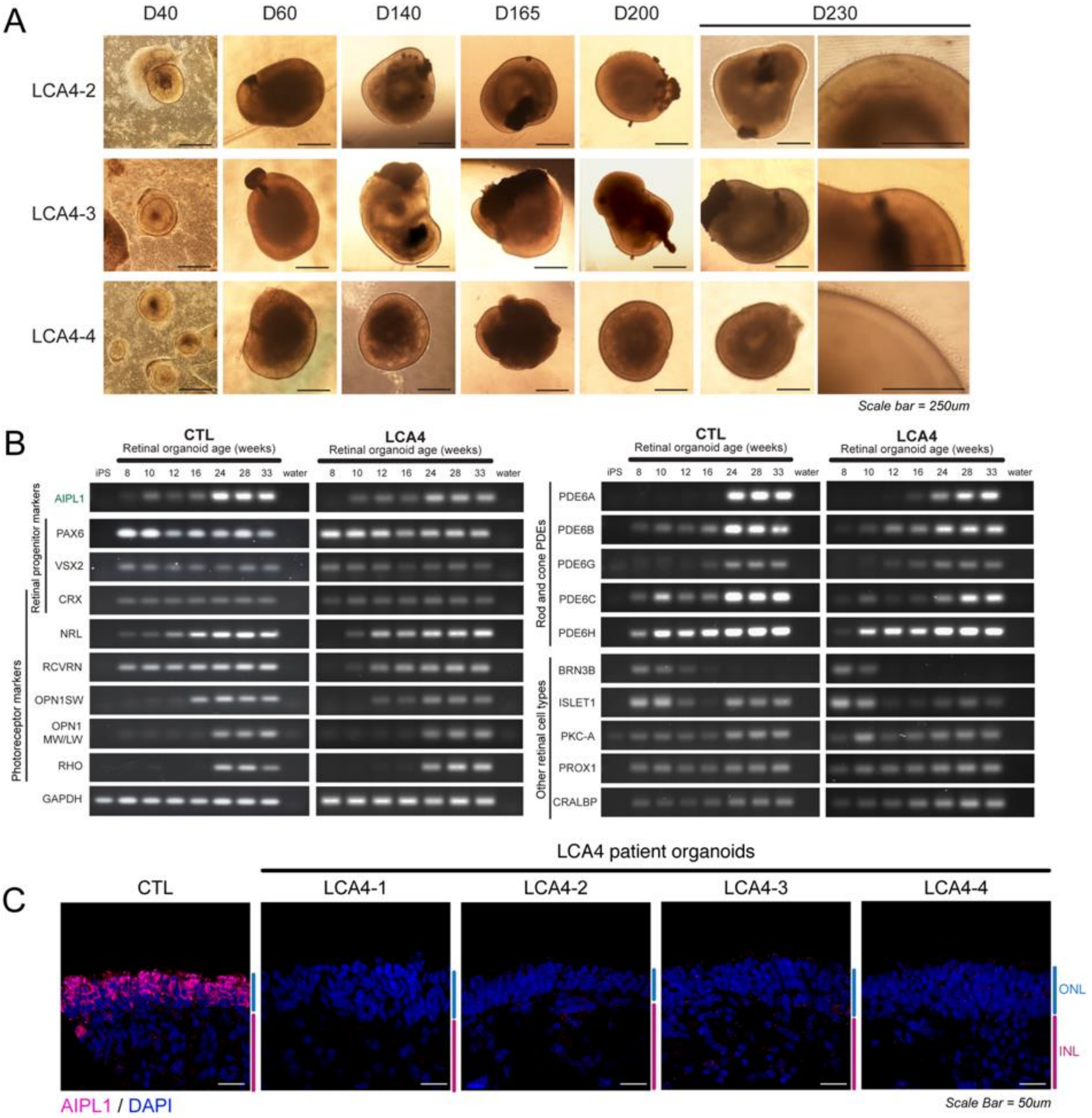

Fig. S4: Analysis of AIPL1 translational readthrough levels in LCA4-1 ROs dosed with varying amounts of PTC124, related to Fig. 5

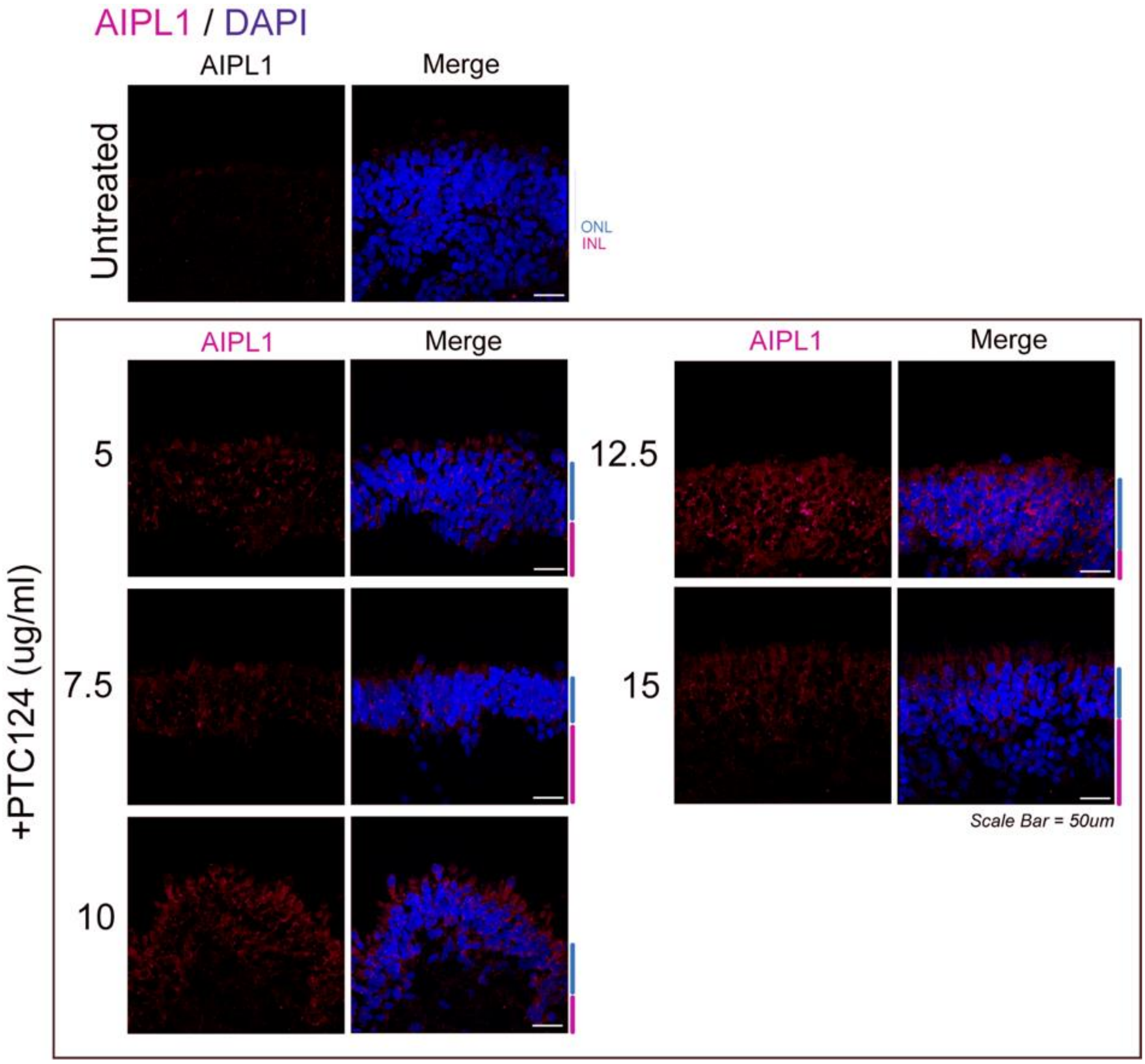

Fig. S5: Characterisation of c.834G>A, p.W278X CRISPR-Cas9 HDR-repaired LCA4-1 isogenic iPSC lines, related to Fig. 5

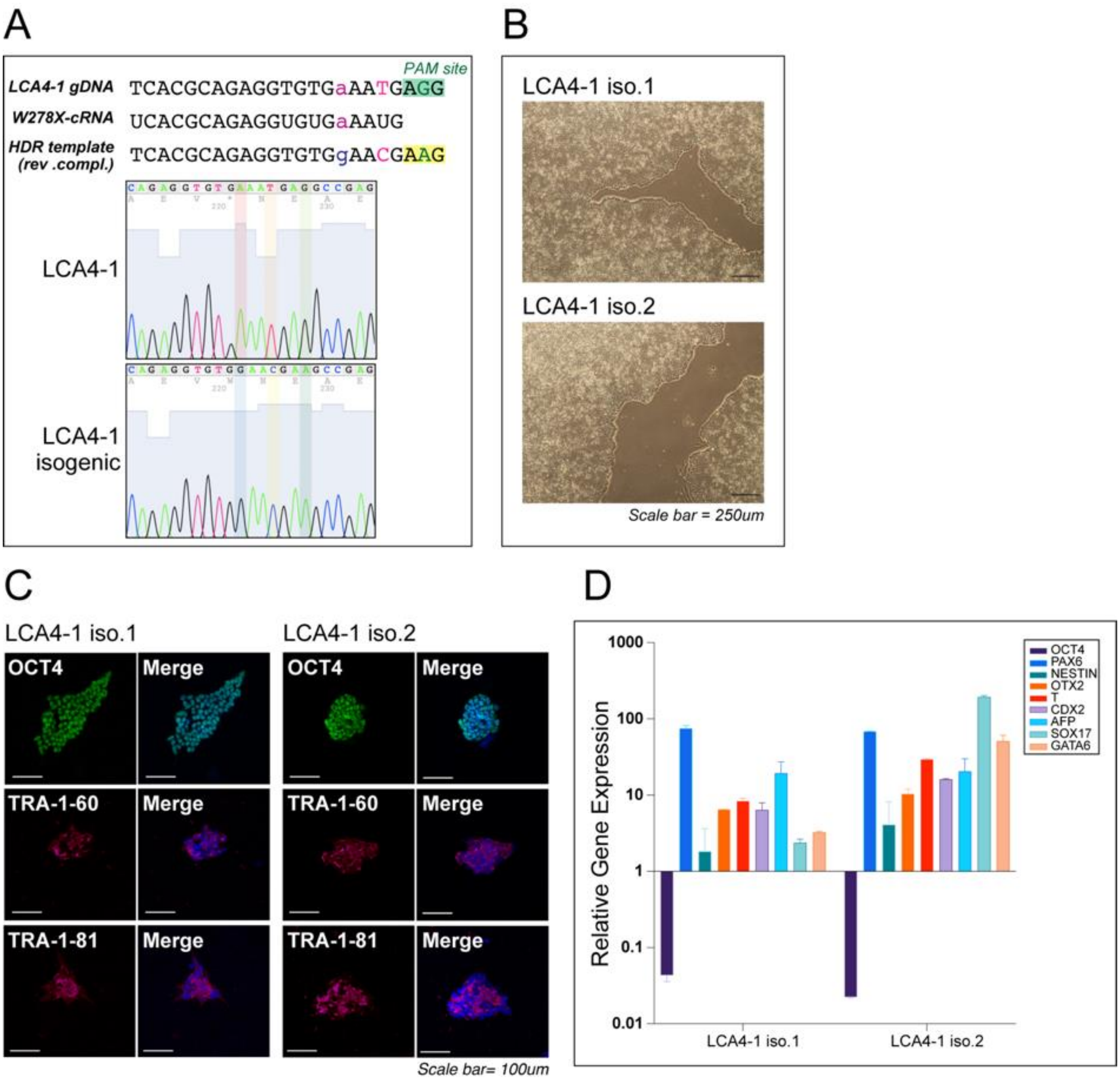

Fig. S6: Further analysis of LCA4-1 ROs with PTC124 treatment: IF and TUNEL assay, related to Figure 5

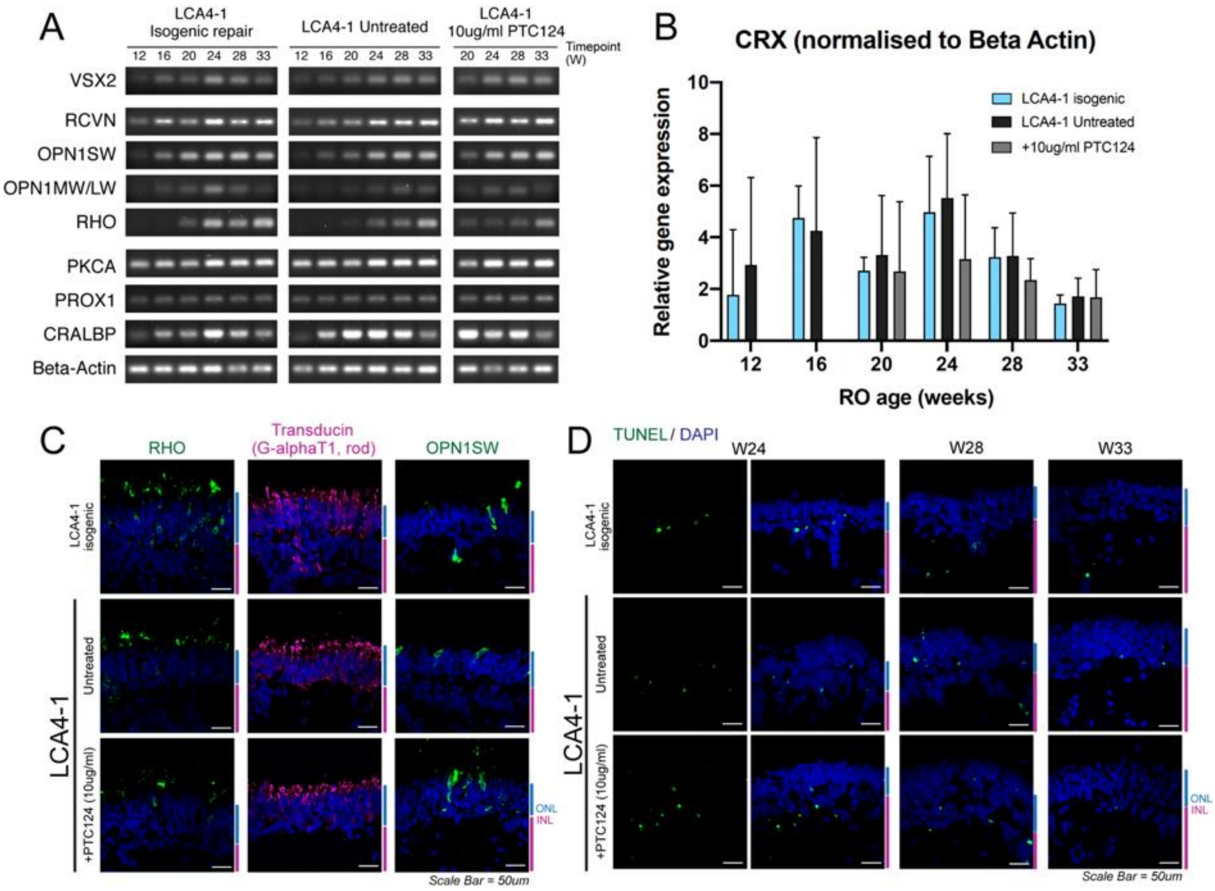

Supplemental Figure Legends

Fig. S1: Molecular and clinical investigation of LCA4 patients, related to Fig. 1

A) Sequence chromatograms of the *AIPL1* gene from control (CTL) and LCA4 patients at the c.834G>A, p.W278X; c.466-1G>C and c.665G>A, p.W222X loci.

B) Patient LCA4-1 (p.W278X homozygote) - OCT image at 3.2 years of age. OCT revealed residual foveal outer retinal structure. Arrows highlight the trace ellipsoid zone in the retina. Patient LCA4-1 had limited visual acuity to perception of light.

C/D) Patients LCA4-2 and LCA4-3 respectively (monozygotic twins; c.834GA, p.W278X; c.466-1G>C compound heterozygotes) - OCT images at 2 years old. OCT revealed residual foveal outer retinal structure. Arrows highlight the trace ellipsoid zone in the retina. Patient LCA4-2 and LCA4-3 had limited visual acuity to perception of light.

E) OCT of a healthy control patient (adult, male, 38y). The scale in grossly comparable with that of the LCA4 patients that did not have axial length measurements.

NFL, nerve fibre layer; GCL, ganglion cell layer; IPL, inner plexiform layer; INL, inner nuclear layer; OPL, outer plexiform layer; HFL, Henle's fiber layer; ONL, outer nuclear layer; ELM, external limiting membrane; EZ, ellipsoid zone; IZ, interdigitation zone; RPE, retinal pigment epithelium.

Fig. S2: Reprogramming of LCA4 patient renal epithelial cells and characterisation of iPSC lines, related to Fig. 1

A) Brightfield images of LCA4 patient renal epithelial cells which were reprogrammed to generate iPSCs. Emerging iPSC colonies were isolated and iPSC lines established from single clones. Scale bars = 250μm.

B) IF analysis of CTL, LCA4-1, LCA-2, LCA-3 and LCA-4 iPSCs for the expression of pluripotency markers (OCT4, Nanog, SSEA4, TRA-1-60, TRA-1-81). DAPI staining is in blue. Scale bars = 100μm.

C) Trilineage analysis of iPSC lines. Real time PCR (qPCR) analysis of iPSC lines subjected to ectodermal (*PAX6, NESTIN, OTX2*)/ mesodermal (*T*)/ endodermal (*CDX2, AFP, SOX17, GATA6*) culture conditions. The pluripotency marker *OCT4* was downregulated in differentiation cultures. All expression levels normalised to

housekeeping beta-actin gene. Values = mean ± SD, from 3 biological replicates (separate differentiations).

Fig. S3: Additional characterisation of LCA4 patient ROs, related to Fig. 2 and 3

A) Brightfield images of developing retinal organoids (D40-230) from LCA4-2, LCA4-3 and LCA4-4 patient iPSC lines. Scale bars = 250μm.

B) Semi-quantitative PCR analysis of developing CTL and LCA4 ROs (W8-W33) for retinal development and retinal lineage markers. 3 biological replicates (individual RO) collected for each time point per differentiation; minimum of two differentiations conducted for each line. Representative PCRs from LCA4-2 ROs samples are shown; the pattern of LCA4 RO gene expression was similar in all LCA4 patient lines.

C) IF of W28 RO sections for AIPL1 (polyclonal antibody raised against recombinant full-length AIPL1). DAPI staining is in blue. ONL and INL regions highlighted at the side of images in blue and magenta. Scale bars = 50μm.

Fig. S4: Analysis of AIPL1 translational readthrough levels in LCA4-1 ROs dosed with varying amounts of PTC124

A) W17 LCA4-1 ROs were treated with 5-15ug/ml PTC124 for 2 weeks, prior to IF analysis. DAPI staining is in blue. ONL and INL regions are highlighted at the side of images in blue and magenta. Scale bars = 50μm.

Fig. S5: Characterisation of c.834G>A, p.W278X CRISPR-Cas9 HDR-repaired LCA4-1 isogenic iPSC lines, related to Fig. 5.

A) Schematic diagram detailing the CRISPR-Cas9 HDR correction strategy of the c.834G>A, p.W278X mutation. 2 additional changes (synonymous) were introduced in the HDR template to remove the PAM site and to prevent re-editing of the locus. Sequence chromatograms of unedited LCA4-1 compared to LCA4-1 isogenic repair cells are shown.

B) Brightfield images of LCA4 isogenic iPSC colonies, isogenic lines 1 (iso.1) and 2 (iso.2). Scale bars = 250μm.

C) IF of LCA4-1 iPSC colonies (iso.1 and iso.2) for pluripotency markers (OCT4, TRA-1-60, TRA-1-81). DAPI staining is in blue. Scale bars = 100μm.

D) Trilineage assay analysis of LCA4 isogenic iPSCs (iso.1 and iso.2). qPCR results showed upregulation of germ-layer specific markers and downregulation of pluripotency marker, *OCT4*. All expression levels normalised to housekeeping beta-actin gene. Values = mean ± SD, from 3 biological replicates (separate differentiations).

Fig. S6: Further analysis of LCA4-1 ROs with PTC124 treatment: IF and TUNEL assay, related to Fig. 5.

A) Semi-quantitative gene expression analyses of LCA4-1 isogenic control, LCA4-1 untreated and PTC124-treated ROs at different developmental timepoints (W12-W33) for genes relating to retinal development and retinal cell lineages. 3 biological replicates (individual RO) per timepoint, minimum of two differentiations conducted for each line.

B) Quantitative PCR analysis of *CRX* levels in LCA4-1 isogenic, LCA4-1 untreated and 10μg/ml PTC124-treated ROs (W12-W33). 3 biological replicates (individual ROs) collected at each timepoint per differentiation, minimum of two differentiations conducted for each line. No significant differences were found between sample types within timepoints. Gene expression levels normalised to beta-actin gene.

C) IF of W24 LCA4-1 isogenic, LCA4-1 untreated and LCA4-1 PTC124-treated ROs for the expression of AIPL1, rhodopsin (RHO), transducin (GT1, rod), and cone opsin (OPN1SW). DAPI staining is in blue. ONL and INL regions are highlighted at the side of images in blue and magenta. Scale bars = 50μm.

D) TUNEL staining of W24/28/33 RO sections showed that apoptosis rates are similar in all RO types at all timepoints with low levels of TUNEL positive cells. DAPI staining is in blue. ONL and INL regions are highlighted at the side of images in blue and magenta. Scale bars = 50μm.

Supplemental Tables

Table S1: List of Antibodies

ICC : antibodies and stains used

Antibody	Species	Antibody info	Catalogue no. / AB	Dilution
AIPL1	Rabbit	J. van der Spuy lab, raised against AIPL1 C-ter		1 / 250
AIPL1	Rabbit	V. Ramamurthy lab		1 / 500
PDE6A	Rabbit	Abcam	ab5659	1 / 1000
PDE6B	Rabbit	Thermo Fisher Scientific	PA1-722	1 / 1500
cGMP	Sheep	BioRad	OBT5055	1 / 100
RETGC1	Rabbit	A.M. Dizhoor lab	/	1 / 4000
Rhodopsin	Mouse	Millipore	4D2	1 / 1000
Cone Arrestin	Mouse	7G6 monoclonal AB	/	1 / 200
RTLN	Mouse	Santa Cruz	C-20 / sc-374056	1 / 200
L/M-Opsin	Rabbit	Millipore	AB5405	1 / 500
S-Opsin	Rabbit	Millipore	AB5407	1 / 500
Transducin, Rod (Gat1)	Rabbit	Santa Cruz	K-20 / sc-389	1 / 100
Recoverin	Rabbit	Millipore		1 / 500
PKCA	Rabbit	Abcam	ab32376	1 / 2000
ISLET1	Rabbit	Santa Cruz - discontinued	/	1 / 50
BRN3B	Rabbit	Santa Cruz - discontinued	/	1 / 10
Vimentin	Rat	R and D systems	MAB2105-SP	1 / 500
PROX1	Rabbit	Sigma Aldrich	AB5475	1 / 2000
OCT4	Rabbit	Abcam	ab19857	1 / 1000
NANOG	Rabbit	Abcam	ab21624	1 / 1000
SSEA4	Mouse	Cell signaling	MC813 / #4755	1 / 1000
TRA-1-81	Mouse	Cell signaling	#4745	1 / 1000
TRA-1-60	Mouse	Cell signaling	#4746	1 / 1000

Secondary Antibodies	Fluorophore	Company	Catalogue no. / AB	Dilution
Donkey anti-mouse IgG	AF488	Thermo Fisher Scientific	A-21202	1 / 1000
Donkey anti-mouse IgG	AF555	Thermo Fisher Scientific	A-31570	1 / 1000
Donkey anti-rabbit IgG	AF555	Thermo Fisher Scientific	A-31572	1 / 1000
Donkey anti-rabbit IgG	AF488	Thermo Fisher Scientific	A-21206	1 / 1000
Donkey anti-sheep IgG	AF488	Molecular Probes	/	1 / 1000
Donkey anti-rat IgG	AF488	Thermo Fisher Scientific	A-21208	1 / 1000

Stain	Fluorophore	Company	Catalogue no. / AB	Dilution
Phalloidin	AF488	Invitrogen / Molecular Probes	A12379	1 : 40 of 200U/ml solution

Table S2: List of Primers – AIPL1 Genotyping / Sanger sequencing

AIPL1 genotyping/sequencing primers

Amplicon	Region	Forward primer	Reverse primer	Expected Size
Genomic	**Exon 1**	ACTGGAAGCAAAGGTGGAT	CCATGCTAAAGTTGAATCTG	526bp
Genomic	**Exon 2**	TGAACTGAGTGAGCTGACCC	GAATAAGTTTGCAGGACTGGCTTTG	428bp
Genomic	**Exon 3**	CATAGTGAGGGAGCAGGATTC	CATGGCTTATGAACCCTCTCG	441bp
Genomic	**Exon 4**	CTTGTCTGTATGCACTTGACCAG	CAGGGAGAAGGTCAGCCATG	426bp
Genomic	**Exon 5**	CGGCTGGGTGGAGACAAG	GAAGTGGCGCTGACTCTGG	369bp
Genomic	**Exon 6**	TTGAGGAAACCGAGGGATGG	CAATCGAACCAGAAGTGACCAGG	582bp
Transcript	**Ex3-5**	TCTACCCCATCCTATCCCG	GGAGAATATCACTGGTGTGCTC	486bp
Transcript	**Ex5-6**	CTGATCCTCAACTACTGCCAG	AGGTGGCTCTGTGGATGA	360bp

Table S3: List of Primers for gene expression analyses – semi-quantitative PCR / qPCR

Real Time PCR and Semi-quantitative PCR

Marker	Forward primer	Reverse primer	Notes
**AIPL1**	ACCGGATCCCGAGTGATCTT	CGATGATGATGTGCATGGGC	qPCR/semi-quant
**VSX2**	GTGGCTACTGGGGATGCAC	TCCTGCTCCATCTTGTCGAG	Semi-quant
**PAX6**	AACGATAACATACCAAGCGTGTC	GTCTGCCCGTTCAACATCCT	Semi-quant
**NRL**	CACTGACCACATCCTCTCGG	GAGGGTTCCCGCTTTACCTC	Semi-quant
**CRX**	TTTGCCAAGACCCAGTACC	GTTCTTGAACCAAACCTGAACC	qPCR/semi-quant
**Recoverin**	ATGAAGTGCTGGAGATCGTC	ATCTTCTCGGCTCGCTTTTC	Semi-quant
**Rhodopsin**	ACCAGCACCCTCTACACCTCTC	AGGACCACAGGGCAATTTC	Semi-quant
**OPN1SW**	CATGTTTGTGCTTTGGAGG	CGAAGGGCTTACAGATGAC	Semi-quant
**MW/LW-OPN1**	CCTATGTGTGTCCTGGAGG	CATCCATCTCTCCCAGGAAATG	Semi-quant
**RETGC1**	ACTGTCCCTCTGAAGGCAG	CGTCATAGATGGTGCCAAAG	qPCR/semi-quant
**PDE6A**	TAACGTCCCCAACACAGAGG	CCACCACATCCTTCCCATTC	qPCR/semi-quant
**PDE6B**	GACGTGTGGTCTGTGCTGAT	CTTGCCGTGGAGGATGTAGTC	qPCR/semi-quant
**PDE6G**	AAGCAGCGACAGACCAGG	TGTGATGTCTGTTCCCAGGC	Semi-quant
**PDE6C**	GTCACTAAGAACCTGCTGGCAACC	AAAGACCTCTTCATCCTGTTTGG	Semi-quant
**PDE6H**	GAGGCAGACTCGCCAATTC	GTGGCTGAATGCCTCCCA	Semi-quant
**PKCA**	GTCCACAAGAGGTGCCATGAA	AAGGTGGGGCTTCCGTAAGT	Harvard Primer Bank / Semi-quant
**ISL1**	GCGGAGTGTAATCAGTATTTGGA	GCATTTGATCCCGTACAACCT	Harvard Primer Bank / Semi-quant
**CRALBP**	AAGCTGGCTACCCTGGTGT	TGAAGCAATATGCCTGCAAGA	Harvard Primer Bank / Semi-quant
**BRN3B**	CTCGCTCGAAGCCTACTTTG	GACGCGCACCACGTTTTTC	Harvard Primer Bank / Semi-quant
**PROX1**	TGAAGACCTACTTCTCCGAC	GACGTGCGTACTTCTCCATC	Semi-quant
**OCT4**	TTTGCCAAGCTCCTGAAGCA	AAGGGCCGCAGCTTACACAT	qPCR
**PAX6**	GCGGTGAGAAGTGTGGGAAC	GCCCGTTGACAAAGACACCA	qPCR
**Nestin**	TCAGATGTGGGAGCTCAATCG	GCTCTTCAGCCAGGTTGTCG	qPCR
**OTX2**	CGCAGTCAATGGGCTGAGTC	ACCGGGTCTTGGCAAACAGT	qPCR
**Brachyury (T)**	CCTTCAGCAAAGTCAAGCTCACC	TGAACTGGGTCTCAGGGAAGCA	qPCR
**CDX2**	TCCTGGACAAGGACGTGAGC	CGCGTAGCCATTCCAGTCCT	qPCR
**AFP**	TGAGCACTGTTGCAGAGGAG	TTGTTTGACAGAGTGTCTTGTTGA	qPCR
**SOX17**	GGATACGCCAGTGACGACCA	CTCGTCCTTAGCCCACACCA	qPCR
**GATA6**	CTGAACGGGACGTACCACCA	GTCTGGATGGAGCCGCAGTT	qPCR
**GAPDH**	CCCCACCACACTGAATCTCC	GGTACTTTATTGATGGTACATGACAAG	qPCR/semi-quant
**Beta Actin**	CCAACCGCGAGAAGATGA	CCAGAGGCGTACAGGGATAG	qPCR/semi-quant

Table S4: List of sgRNAs and ssODN Sequences – CRISPR-Cas9 HDR

List of sgRNAs and ssODN Sequences – CRISPR/Cas9 HDR

Combination	sgRNA sequence	ssODN template sequence* (* denote phosphorothioate (PS) bonds)
1	TCACGCAGAGGTGTGAAATG	T*C*CAGCAGCCTCAGCTCCCTGCGCACCGCCTTCTGCATGGACGGCTCCAGCTCCAGCACTTTCTGGAGGTCCGCCTT GGCCTCGGCTTCGTTCCACACCTCTGCGTGAGCCCGGGCACGCACGTA*G*T
2	AGAGGTGTGAAATGAGGCCG	C*G*GTTCTCCAGCAGCCTCAGCTCCCTGCGCACCGCCTTCTGCATGGACGGCTCCAGCTCCAGCACTTTCTGGAGGTC CGCCTTGGCCTCGGCCTCGTTCCACACCTCAGCGTGAGCCCGGGCACG*C*A

The combination of sgRNA1 and ssoDN template1 was able to trigger CRISPR-Cas9 HDR of the p.W278X locus with an estimated efficiency of editing at the p.W278X locus of approximately 30%, with HDR enhancer (as determined by TIDER analysis (http://shinyapps.datacurators.nl/tider/)). This was used to establish LCA4-1 isogenic iPS lines.

No editing was detected with sgRNA2 and ssODN template2.

Table S5: List of Primers – CRISPR-Cas9 HDR Predicted Off-Target sites.

AIPL1-W278X locus CRISPR: off-target analysis

Program	Region	Site of potential off-target editing	Sequence of potential binding site*	Bulge size*	Mismatches*	Forward primer	Reverse primer
CASOFFFINDER	**Site 1**	Chr.14, NC_000014.9: 67687177 to 67687198	**crRNA: TCACGCAGAGGTGTGAAATGNGG**	1	1	CACCTAAAGCATGCATCTCC	CTAAGAGCAATGGGAAACGG
			**DNA: TCA-GCAGAGGTGaGAAATGAGG**				
CASOFFFINDER	**Site 2**	Chr.8, NC_000008.11 : 142474973 to 142474996	**crRNA: TCAC-GCAGAGGTGTGAAATGNGG**	1	2	AGGCCTTGATCACCACTG	CAGGCTCCGTCCAGTTTC
			**DNA: TCACAGCtGgGGTGTGAAATGTGG**				
CASOFFFINDER	**Site 3**	Chr.3, NC_000003.12 : 172174110 to 172174131	**crRNA: TCACGCAGAGGTGTGAAATGNGG**	1	2	GCGAACTGAGAATCCATTCATG	CCAAATATGCTTGAATGCCTTG
			**DNA: gCAtGCAGAGGTGTGAAA-GTGG**				
CASOFFFINDER	**Site 4**	Chr.4, NC_000004.12 : 186616865 to 186616886	**crRNA: TCACGCAGAGGTGTGAAATGNGG**	1	2	GTTGGATGCAACTGGAGTC	TCAATGGGACCATCATTTGG
			**DNA: TaA-GCAGAGGTGTtAAATGGGG**				
CASOFFFINDER	**Site 5**	Chr.7, NC_000007.14 : 146187291 to 146187314	**crRNA: TCAC-GCAGAGGTGTGAAATGNGG**	1	2	CCAACTCTGGTAAATGTGAGTAG	CTCTTCTTCGGCTCTCAGTG
			**DNA: TCACAGCAGAGGTGTtAcATGTGG**				
OFFSPOTTER	**Site 6**	Chr.10, NC_000010.11 : 131402426 to 131402448	**crRNA: TCACGCAGAGGTGTGAAATGNGG**	0	3	TCTTAATATTGATGCCAGCCTG	AGCCAGTTCTCCTGTTGAT
			**DNA: gCggGCAGAGGTGTGAAATGAGG**				
OFFSPOTTER	**Site 7**	Chr.X, NC_000023.1: 25154339 to 25154361	**crRNA: TCACGCAGAGGTGTGAAATGNGG**	0	3	AATGTACTTCACTACACATCGTC	GTCAACTCAACATTTCTCTCTTC
			**DNA: aCAaGCAGAGaTGTGAAATGTGG**				
OFFSPOTTER	**Site 8**	Chr.4, NC_000004.12 : 186616865 to 186616887	**crRNA: TCACGCAGAGGTGTGAAATGNGG**	0	3	Same as for Site 4	Same as for Site 4
			**DNA: TtAaGCAGAGGTGTtAAATGGGG**				
OFFSPOTTER	**Site 9**	Chr.4, NC_000004.12 : 21759877 to 21759899	**crRNA: TCACGCAGAGGTGTGAAATGNGG**	0	3	CCACCTGTCCTTGTAAACTG	GTTGGAGACCTGTAGTTCTTTC
			**DNA: TCAaGCAGAGGTGaaAAATGAGG**				
OFFSPOTTER	**Site 10**	Chr.12, NC_000012.12 : 59076201 to 59076223	**crRNA: TCACGCAGAGGTGTGAAATGNGG**	0	4	GCTGAAATAATCATGAAGGCATG	CAGATATAGCTGAATCCAGGTAC
			**DNA: gaAaGCAGAGaTGTGAAATGCGG**				

***Bulges and base pair mismatches are highlighted in red**

Supplemental Experimental Procedures

Clinical Data and Patient Imaging

Medical notes and clinical images were reviewed. This included results of comprehensive ophthalmic clinical assessment, including dilated fundoscopy and age-appropriate visual acuity assessments, and electrophysiological testing. Optical coherence tomography (OCT) imaging was reviewed. OCT was acquired with handheld Bioptigen spectral domain OCT (Leica Microsystems, Research Triangle Park, NC, USA). Trans-foveal horizontal scan of a healthy adult was acquired using Heidelberg Spectralis OCT (Heidelberg Engineering, Heidelberg, Germany).

Urine Collection, Cell Isolation and Expansion of Renal Epithelial Cells

Urine was collected from LCA patients with mutations in the *AIPL1* gene. Collection, isolation, and expansion of renal epithelial cells was performed similarly to described previously (Hildebrand et al., 2016; Zhou et al., 2012). Briefly, urine samples (volumes varying from 15mls to 80mls) were collected in sterile tubes and centrifuged at 400g for 10min at room temperature. After discarding the supernatant, pellets were washed with 10mls of PBS containing 500ng/ml ampothericin B and 100 U/ml penicillin/streptomycin and centrifuged again at 400g for 10min. Pellets were resuspended in 2mls of Primary Medium consisting of DMEM/Ham's F-12 nutrient mix (1:1) (ThermoFisher Scientific), with 10% of fetal bovine serum (FBS), Renal Cell Growth Medium (REGM) SingleQuot kit supplements (Lonza), 2.5µg/ml amphotericin B, and 100 U/ml of penicillin/streptomycin. The cells were seeded into one well of a 12-well plate coated with 0.1% gelatin. One ml of Primary Medium was added to the well at 24h, 48 and 72h without removing any media. Renal epithelial cells routinely appear within the first 3-5 days of culture. 96h post seeding, most of the medium was removed and replaced with Proliferation Medium, consisting of (1:1 mixture of Renal Cell Growth Medium (REBM) medium supplemented with REGM SingleQuots (Lonza) and DMEM high glucose (ThermoFisher Scientific) supplemented with 10% FBS, 1% GlutaMAX, 1% non-essential amino acids (NEAA), 100 U/ml penicillin/streptomycin. Subsequently, half of the culture medium was changed every day. Once cell density reached 90% (between 14–28 days after urine collection), cells were split 1:4 using 0.25% Trypsin-EDTA, (ThermoFisher Scientific) and expanded for a maximum of four passages.

Reprogramming, Culture, and Characterization of iPSCs

Renal epithelial cells passaged fewer than 3 times were used for iPSC generation as described previously (Xue et al., 2013). Briefly, 5 x 10^5 renal epithelial cells were trypsinized (0.25% Trypsin-EDTA, ThermoFisher Scientific) and electroporated with integration-free episomal plasmids pCXLE-hOCT3/4-shp53-F, pCXLE-hUL, and pCXLE-hSK (Addgene) and miRNA 302/367 plasmid (Gift from Dr J. A.Thomson, Regenerative Biology, Morgridge Institute for Research, Madison, Wisconsin, USA) using the Amaxa™ Basic Nucleofector™ Kit for primary mammalian epithelial cells, program T-020 (Lonza). Electroporated cells were plated onto geltrex-coated 12 well plates and cultured in E8 media (Gibco), which was changed every day. The iPSC colonies were picked at around day 14 and expanded in E8 media on geltrex-coated 6 well plates to establish individual iPSC lines. iPS cultures were routinely passaged using Versene (Gibco). Pluripotency of the isolated iPSC lines was confirmed by immunofluorescence (IF), using iPSC-specific antibodies (Supplementary Table 1**)**. iPSC cultures were grown in 8-well permanox chamber slides (ThermoFisher Scientific) and fixed in 4% PFA:PBS for 15 minutes at RT prior to IF. Genomic DNA was extracted for amplification of the *AIPL1* gene and PCR products were Sanger sequenced to confirm the presence of *AIPL1* mutations in the different patient lines (see Supplementary Table 2 for primer sequences). To confirm trilineage differentiation potential, iPSCs were differentiated into the three germ layers using the StemDiff™ Trilineage Differentiation Kit (STEMCELL Technologies) according to manufacturer instructions, and the resultant tissues analysed for the expression of key germ layer markers by real time PCR (Supplementary Table 3). iPSC lines from well-characterised controls (CTL) (commercial control human dermal fibroblasts obtained from ATCC (Parfitt et al., 2016) were expanded in parallel.

CRISPR-Cas9 Homology-Directed Repair (HDR) of AIPL1 p.W278X allele

20bp guide RNAs (gRNAs) were designed around the locus (NGG PAM) using Benchling software. 127bp single stranded oligo deoxynucleotide (ssODN) repair templates (antisense to the target strand) were designed for HDR, with additional synonymous changes introduced to remove the PAM site/prevent re-editing of the locus (Supplementary Table 4). CRISPR RNA (crRNA) and ssODN templates (ultramer oligos with phosphorothioate (PS) modification of the two 5’ and 3’

nucleotides) were from IDT. iPSC cultures were grown in Stemflex (Gibco) supplemented with 10μM ROCK inhibitor Y-27632 (StemCell Technologies) for 2 hours prior to single cell dissociation with TrypLE (Gibco). 2 x 10^5 iPS cells/sample were nucleofected with 130pmol of crRNA:tracrRNA duplex (Cas9 nuclease V3 tracrRNA) (IDT) complexed with 125pmol of Alt-R Cas9 V3 enzyme (61 uM) (IDT), 200pmol ssODN template and 120pmol Alt-R Electroporation enhancer (IDT), using a P3 Primary Cell 4D-Nucleofector X Kit S (Lonza). Nucleofections were conducted both with or without HDR enhancer (IDT). Cells were plated onto rhLaminin-521 (ThermoFisher Scientific) coated wells (24-well plates) in Stemflex + ROCKinhibitor, and cultured thereafter in Stemflex until single cell cloning to isolate correctly edited iPSC clones. 3 correctly edited clones (homozygous correction of p.W278X) were established from LCA4-1 iPSC using sgRNA 1 and ssODN repair template 1 (Supplementary Table 4), of which 2 (LCA4-1 isogenic 1 and 2) were further characterised with respect to pluripotency markers and trilineage potential (Supplementary Figure 4). Potential genomic off-target sites were identified using Cas-OFFinder (http://www.rgenome.net/cas-offinder/ - Bae et al., 2014) and Offspotter (https://cm.jefferson.edu/Off-Spotter/). Primers were designed to amplify the top 9 regions and products from LCA4-1 and the 2 isogenic lines were subject to Sanger sequencing – no changes were detected in the isogenic lines (Supplementary Table 5). LCA4-1 isogenic line 2 was used for all experiments.

Differentiation of Retinal Organoids (ROs)

iPSC cultures were dissociated with Versene (Gibco) and cell clumps collected, washed twice with PBS, and seeded at high density in E8 media in 6 well plates coated with Geltrex (Gibco). iPSC colonies were grown until 90-95% confluency, then Essential 6™ media (Gibco) was added for 2 days (Day 1 and Day 2 of differentiation) followed by a neural induction period in Neural Induction Media (Advanced DMEM/F12 (1:1, Gibco), 1% non-essential amino acids (Gibco, NEAA), 1% N2 Supplement (Gibco), 1% GlutaMAX (Gibco) and 100 U/ml penicillin/streptomycin (Gibco)). Some cultures were supplemented with human recombinant bone morphogenetic protein 4 (rhBMP4) to improve neural induction efficiency; on Day 6, media was supplemented with 1.5nM BMP4 (R&D Systems), and half media changes were carried out every other day until Day 16 to dilute the rhBMP4. Around week (W) 6, neuro-retinal vesicles (NRVs) were manually excised using 21G needles/scalpel blades and grown in low-

binding 96 well plates (96 well Nunc Sphera Round Bottom Plates, ThermoFisher Scientific) in Retinal Differentiation Media (DMEM/F12 nutrient mix (3:1 ratio, Gibco), 10% fetal bovine serum (FBS, Gibco), 2% B27 supplement (without vitamin A), 100uM taurine, 2mM GlutaMAX and 100U/ml penicillin/streptomycin), with media changes every 2 days. At W10, cultures were supplemented with 1uM retinoic acid (RA), and ROs were transferred into low binding 24-well plates. At W12, the cultures were supplemented with 1% N2 and the RA concentration was reduced to 0.5uM. At W14 (D100), B27 and RA were removed from the medium.

Supplemental References

Bae, S., Park, J., and Kim, J.-S. (2014). Cas-OFFinder: a fast and versatile algorithm that searches for potential off-target sites of Cas9 RNA-guided endonucleases. Bioinforma. Oxf. Engl. 30, 1473–1475. https://doi.org/10.1093/bioinformatics/btu048.

Hildebrand, L., Rossbach, B., Kühnen, P., Gossen, M., Kurtz, A., Reinke, P., Seemann, P., and Stachelscheid, H. (2016). Generation of integration free induced pluripotent stem cells from fibrodysplasia ossificans progressiva (FOP) patients from urine samples. *Stem Cell Res*. *16*, 54–58. https://doi.org/10.1016/j.scr.2015.11.017.

Parfitt, D.A., Lane, A., Ramsden, C.M., Carr, A.-J.F., Munro, P.M., Jovanovic, K., Schwarz, N., Kanuga, N., Muthiah, M.N., Hull, S., et al. (2016). Identification and Correction of Mechanisms Underlying Inherited Blindness in Human iPSC-Derived Optic Cups. *Cell Stem Cell 18*, 769–781. https://doi.org/10.1016/j.stem.2016.03.021.

Xue, Y., Cai, X., Wang, L., Liao, B., Zhang, H., Shan, Y., Chen, Q., Zhou, T., Li, X., Hou, J., et al. (2013). Generating a non-integrating human induced pluripotent stem cell bank from urine-derived cells. *PloS One 8*, e70573. https://doi.org/10.1371/journal.pone.0070573.

Zhou, T., Benda, C., Dunzinger, S., Huang, Y., Ho, J.C., Yang, J., Wang, Y., Zhang, Y., Zhuang, Q., Li, Y., et al. (2012). Generation of human induced pluripotent stem cells from urine samples. *Nat. Protoc. 7*, 2080–2089. https://doi.org/10.1038/nprot.2012.115.
